# Genome-Wide Association Mapping in the Global Diversity Set Reveals New QTL Controlling Root System and Related Shoot Variation in Barley

**DOI:** 10.3389/fpls.2016.01061

**Published:** 2016-07-19

**Authors:** Stephan Reinert, Annika Kortz, Jens Léon, Ali A. Naz

**Affiliations:** Institute of Crop Science and Resource Conservation, Chair of Plant Breeding, University of BonnBonn, Germany

**Keywords:** association mapping, barley diversity, fibrous rooting, QTL, root and shoot, drought tolerance

## Abstract

The fibrous root system is a visible sign of ecological adaptation among barley natural populations. In the present study, we utilized rich barley diversity to dissect the genetic basis of root system variation and its link with shoot attributes under well-water and drought conditions. Genome-wide association mapping of phenotype data using a dense genetic map (5892 SNP markers) revealed 17 putative QTL for root and shoot traits. Among these, at 14 loci the preeminence of exotic QTL alleles resulted in trait improvements. The most promising QTL were quantified using haplotype analysis at local and global genome levels. The strongest QTL was found on chromosome 1H which accounted for root dry weight and tiller number simultaneously. Candidate gene analysis across the targeted region detected a crucial amino acid substitution mutation in the conserved domain of a WRKY29 transcription factor among genotypes bearing major and minor QTL alleles. Similarly, the drought inducible QTL QRdw.5H (5H, 95.0 cM) seems to underlie 37 amino acid deletion and substitution mutations in the conserved domain of two related genes CBF10B and CBF10A, respectively. The identification and further characterization of these candidate genes will be essential to decipher genetics behind developmental and natural adaptation mechanisms of barley.

## Introduction

Natural populations of crop plants have evolved vital traits which play fundamental role in their production and adaptation (Annicchiarico et al., 2013; Vadez, 2014). The expression of these traits and adaptive strategies is modulated by complex network of genetic and environmental components (Russell et al., 2014). Therefore, a detailed genetic dissection and understanding of these traits using natural genetic resources is essential to uncover new breeding leads and their direct utility in improving agronomic traits and drought stress tolerance. The improved performance of crop plants under drought appeared as one the most important question of current and future challenges of plant breeding with respect to climate change scenario (Pennisi, 2008; Comas et al., 2013).

Roots and their architecture are seen as the most important plant organ for crop productivity and adaptation to drought stress due to their versatile ability in capturing water and nutrients. Furthermore, roots are the prime organs that sense and respond to water deficit conditions (Naz et al., 2012; Vadez, 2014). Especially, deeper and more profuse root systems increase the drought tolerance of crops like rice, wheat and barley (Chloupek et al., 2010; Uga et al., 2013). For instance, Uga et al. discovered DEEPER ROOTING 1 (*Dro1*) gene which mediates fibrous rooting in rice and established gene bearing near isogenic lines (NILs; Uga et al., 2013). *Dro 1-*NIL exhibited a significant increase in yield performance under drought conditions due to increased drought avoidance by deep rooting compared to control genotype IR64.

Barley root system comprises of two components: seminal and nodal roots (Wahbi, 1995). Seminal roots develop in the post-embryogenesis from embryo’s radical whereas nodal roots are initiated through the base of each established tiller later in plant development (Wahbi, 1995). This process continues for at least eight weeks depending upon the ability of nutrients and suitable environmental conditions (Lancashire et al., 1991). The development of each tiller above ground consequently increases the number of nodal roots below ground because of their location close to soil. Both seminal and nodal roots develop lateral roots and water sucking organs, the root hairs (Naz et al., 2012; Smith and De Smet, 2012). This peculiar developmental scheme is the rule in cereal crops like wheat and barley suggesting two parallel mechanisms influencing root system variation; i) the inherent seminal rooting ability and ii) shoot dependent nodal root initiation. The latter mechanism seems more complex because it is still unclear if more tillering is the cause of more nodal rooting or if there exists positive feedback in which an increase in nodal rooting facilitates more shoot development by the acquisition of more water and nutrients. Several studies were made to find the interplay of root and shoot dependency in cereals. For instance, Narayanan and Vara Prasad found a close relationship of root and shoot traits, especially for shoot dry weight (Sdw) and tiller number (Til) to most root traits in a spring wheat association panel comprising 250 genotypes (Narayanan and Prasad, 2014). Moreover, Canè et al. detected in a GWAS analysis of 183 durum elite accessions 15 overlapping QTL for root and agronomic traits and/or grain yield in two or more environments (Canè et al., 2014). Recently, Lou et al. performed in depths genetic analysis of deep rooting in rice and predicted the role of auxin associated genes in mediating different root attributes of rice (Lou et al., 2015).

Barley natural diversity presents manifold genetic resources in the form of wild accessions, landraces and cultivars. Interestingly, these genetic resources were evolved over time by passing the bottlenecks of adverse climatic conditions. Hence, in this process these resources established novel adaptive measures which have given them fitness advantage for a particular environment (Russell et al., 2014). The wild barley accessions reveal immense variations in root system and its architecture (Grando and Ceccarelli, 1995; Nevo and Chen, 2010). Naz et al. (2012) as well as Grando and Ceccarelli found dramatic difference of root and shoot traits across wild and cultivated barley where wild barley accessions, adapted to the desert conditions of Middle East, developed highly fibrous root system (Grando and Ceccarelli, 1995; Uga et al., 2013). It is believed that highly fibrous and deep root system in wild barley accessions adapted to semi-desert conditions may offer a fitness advantage under drought. Furthermore, this diversity revealed stunning differences for root and shoot traits as well as a clear interplay of root and shoot development in barley. Although, natural diversity of barley showed significant variations, very little has been done to employ these valuable genetic resources in breeding to improve root architecture and drought stress tolerance in barley. The biggest challenge behind this was the poor genetic understanding of root and related shoot traits primarily due to difficulty in large scale root phenotyping. This scenario thus demands a comprehensive analysis of root system variation and its link with shoot development across the barley global diversity set to find novel breeding leads for the improvement of root and adaptive traits among modern varieties.

In the present study, we performed genome-wide association mapping for root and shoot traits using a unique barley diversity set adapted to different environmental conditions across the world. The diversity set was established based on modern cultivars, landraces and wild accessions for in depths analyses of broad spectrum genetic resources to discover essential breeding leads. A highly dense genetic map based on SNP markers was utilized to understand the genetic basis of root, shoot traits as well as their putative interplay under control and drought stress conditions. Interestingly, the above mentioned genetic resources can easily be hybridized with each other offering an advantage for a straight forward transfer of valuable exotic alleles from landraces and wild barley accessions to cultivated varieties.

## Results

### Population Structure Analysis

Population structure was calculated in order to see the structural pattern of global barley population. The best K value detection implemented in CLUMPAK revealed three distinct sub-clusters within the population (Supplementary Figure S1). Therefore, kinship and PCA had to be included in association mapping analysis to reduce structural effects during the calculations.

Linkage disequilibrium (LD) was calculated to see the genetic recombination across the chromosomes. This revealed the LD-decay for all chromosomes among all genotypes (Supplementary Figure S2D). The recombination fraction (*r*^2^) of chromosome 7H decreased from 0.17 to <0.1 within 6.7 cM, whereas chromosomes 1H to 6H exhibited *r*^2^ below 0.1. For the purpose of showing differences in genetic recombination due to genomic background of genotypes, we calculated LD for three sub-pops: cultivars, landraces and wild accessions. The cultivated barley revealed the highest recombination fraction across all chromosomes compared to barley landraces and wild types (Supplementary Figure S2A). On the other hand, wild barley sub-pop (Supplementary Figure S2C) showed the lowest recombination fraction, whereas barley landraces (Supplementary Figure S2B) possessed a recombination fraction between cultivated barley and wild barley. Overall, the sub-pops showed a clear pattern of LD-decay for all chromosomes. Furthermore, chromosome 7H revealed for cultivated barley and landraces the highest recombination fraction compared to chromosomes 1H to 6H. Whereas, wild barley sub-pop exhibited equal recombination fraction for chromosomes 1H to 7H compared to cultivars and landraces.

### Trait Variation

The analysis of variance revealed a high diversity among genotypes within the global barley population. Moreover, this population showed highly significant differences between drought and control conditions for all traits. The effect for genotype by treatment was highly significant for most traits except Rl. However, the interaction effect of genotype by year revealed highly significant variations for all five traits. Similarly, the genotype by treatment by year effect showed significant differences for Rdw, Sdw, and Til. The broad-sense heritability (*H*^2^) revealed high coefficients for Rdw (0.62), Rl (0.48) Sdw (0.54), RS (0.66), and the highest heritability for Til (0.90) (Supplementary Table S2).

Mean comparison of trait values showed significant variation in the different environments like control and drought conditions as well as in years 2014 and 2015 (Supplementary Figures S3-S12). Overall, the trait values were reduced significantly under drought stress conditions as compared to control. The population wide mean comparison showed strong differences for Rdw under control and drought conditions with 9.7 and 5.1 g, respectively, in 2014 (Supplementary Figure S3) as well as 6.2 and 3.3 g, respectively, in 2015 (Supplementary Figure S4). Similarly, we observed strong differences for Sdw, Til, and RS under drought and control conditions (Supplementary Figures S7-S12). The trait Rl revealed least mean differences across drought stress and control blocks (Supplementary Figures S5-S6).

In order to see the relationship of root and shoot traits, Pearson correlation was calculated for Rdw, Rl, Sdw, Til, and RS under control and drought conditions (Supplementary Table S3). For Rdw and RS (0.80), the correlation revealed the highest significant positive correlation among all traits under control conditions. Furthermore, Sdw and RS revealed the highest negative correlation under control conditions (-0.53). Rdw and Rl (0.11) showed no correlation under control conditions. Under drought conditions, Rdw and Til showed the strongest positive correlation (0.49). Moreover, the strongest negative correlation under drought conditions was observed for RS and Sdw (-0.47).

### GWAS-QTL Detection and Quantification

GWAS analysis revealed 17 significant marker by trait associations for five analyzed root and shoot traits within the global barley population (**Table 1**). A QTL map showing the associated and flanking SNP markers across the chromosomes is presented Supplementary Figure S13.

**Table 1:** List of significant QTL regions for root and shoot traits with marker information and trait effect of particular allele analyzed in the global population.

Trait	QTL	Marker	Effect	Pos (cM)	Flanking region	LOD	Var (%)	Major/Minor	Major	Het	Minor	RP (%)
**Rdw**	QRdw.1H	BOPA1_7381-1292	M	1H (122.17)	122.09-122.17	11.57	13.81	G/A	5.84	4.09	7.48	82.76
	QRdw.2H	SCRI_RS_918	MxT	2H (106.80)	106.79-107.97	17.77	18.59	T/C	7.85	7.15	5.60	40.18
	QRdw.3H	BOPA1_ABC13678-1-2-369	M	3H (122.59)	120.68-124.54	14.54	20.76	A/G	5.90	6.70	8.39	42.20
	QRdw.5H	BOPA2_12_30850	M / MxT	5H (95.00)	94.44-99.93	26.20	24.93	G/A	6.96	7.52	9.48	36.16
**Rl**	QRl.5H	SCRI_RS_159430	M	5H (93.40)	91.16-93.40	16.14	14.29	T/C	46.00	47.50	48.67	5.80
	QRl.7H	SCRI_RS_157337	M	7H (3.82)	3.82-3.82	15.37	10.12	C/T	47.33	45.75	44.00	7.57
**Sdw**	QSdw.2H.a	BOPA2_12_20878	M	2H (58.99)	54.32-62.46	40.70	33.66	A/G	20.80	26.62	15.88	67.65
	QSdw.2H.b	SCRI_RS_918	MxT	2H (106.80)	104.15-111.26	32.05	27.85	T/C	17.61	19.56	22.85	29.76
	QSdw.4H	SCRI_RS_167844	M	4H (48.65)	48.65-53.47	22.66	19.86	G/A	21.24	16.83	16.42	29.32
**Til**	QTil.1H	BOPA1_7381-1292	M	1H (122.17)	118.34-127.09	102.61	53.20	G/A	10.00	14.50	15.00	50.00
	QTil.2H	SCRI_RS_218303	M	2H (53.26)	48.44-58.05	39.55	35.91	C/T	11.00	19.50	14.00	77.27
	QTil.7H.	BOPA1_497-386	M/MxT	7H (57.93)	52.97-61.47	35.99	28.84	G/A	11.00	13.00	20.00	81.82
**RS**	QRS.2H	SCRI_RS_918	MxT	2H (106.80)	106.80-106.80	14.15	15.08	T/C	0.42	0.38	0.30	38.33
	QRS.3H	BOPA2_12_11482	M/MxT	3H (52.62)	51.14-52.62	17.76	13.54	A/C	0.36	0.81	0.37	125.00
	QRS.4H	BOPA1_ABC14026-1-2-168	M	4H (51.40)	48.65-51.40	13.94	15.88	A/G	0.34	0.36	0.42	23.53
	QRS.5H	BOPA2_12_30850	M/MxT	5H (95.00)	93.40-95.00	66.09	29.30	G/A	0.35	0.38	0.55	57.14
	QRS.7H	SCRI_RS_152299	M/MxT	7H (61.47)	57.93-61.47	11.19	12.86	C/T	0.34	0.39	0.39	14.71

*Trait: RDW, Root dry weight; RL, Root length; SDW, Shoot dry weight; Til, No of tiller; RS, Root-shoot ratio; M, main effect; MxT, marker by treatment effect; Pos, cM position on chromosome; Chr, Number of chromosome; LOD, LOD score; Var (%), genetic variation explained by a single QTL; Major/Minor, Major allele and minor allele; Major/Het/Minor, Phenotypic effect of the homozygous major allele, heterozygous allele and minor allele; RP (%), Relative performance of positive allele compared to negative allele.*

#### Root Dry Weight (Rdw)

**FIGURE 1:**
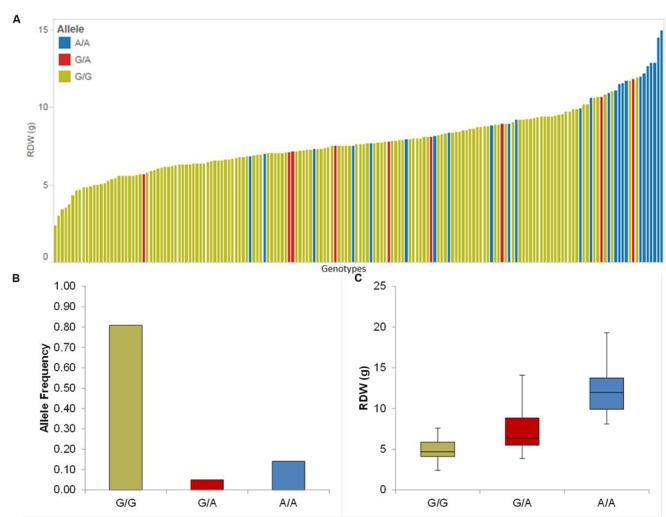
**Quantification of allele based trait effect of QRdw.5H. (A)** Pin plot analysis based on allelic effects for Rdw across the whole population. Genotypes are ordered based on their average Rdw in 2014 and 2015. **(B)** Allele frequency at QTL QRdw.5H. **(C)** Whisker plot for 10 randomly selected genotypes per allele to quantify the trait effect of the particular allele, except heterozygous allele. Yellow: Major allele; Red: Heterozygous allele; Blue: Minor allele.

We detected four putative QTL for Rdw located on chromosomes 1H, 2H, 3H, and 5H. The summary statistics as well as the relative performance (RP) for all QTL is presented in **Table 1**. Among these, the strongest QTL (QRdw.5H) based on LOD was located on chromosome 5H between 94.44 and 99.93 cM, where the minor allele affects the RP by about 36.16%. Another notable QTL was QRdw.1H on chromosome 1H between 122.09 and 122.17 cM which influenced the RP positively by 82.76%. The effect of the strongest QTL (QRdw.5H) was visualized in a pin plot to see the allele-wise differences of the phenotype among the whole population. The genotypes carrying the homozygous allele A/A of QRdw.5H exhibited the maximum phenotypic effect. On the other hand genotypes bearing the homozygous allele G/G showed a moderate phenotypic affect compared to homozygous A/A allele (**Figure 1A**). The individual genotypes on x-axis of this pin plot can be identified in Supplementary Table S4. Later on, we analyzed the allele-wise distribution of QRdw.5H to detect the major (G/G) and minor (A/A) allele (**Figure 1B**; Supplementary Figure S21). Genotypes carrying the minor allele are mostly wild barley accessions. Homozygous A/A allele is revealing the highest Rdw (average 13 g) whereas the mean of the homozygous major G/G allele is 5 g. The heterozygous allele showed an average effect in between the homozygous alleles (**Figure 1C**).

For haplotype analysis, we randomly selected 30 genotypes of most promising QTL regions and computed the local and global genetic relatedness at the genome level. Based on the LD analysis we chose a 5 cM area left and right from the particular significant marker for the local comparison. In order to see the genetic background of genotypes possessing homozygous G/G allele and A/A allele we performed the local and global comparison of those haplotypic groups. The local genetic comparison of QRdw.5H for a region between 90.18 and 98.89 cM revealed a sub-pop based relationship of genotypes for the minor allele A/A. A marginal genetic similarity was observed between sub-pop I and sub-pop II after comparing the local genetic composition of both sub-pops. Similarly, the comparison of haplotypic sub-pop II and sub-pop III exhibited a moderate overall genetic relatedness like sub-pop I and sub-pop II. Furthermore, sub-pop I and sub-pop II showed a high genetic diversity among genotypes within each haplotypic sub-pop. In contrast, sub-pop III possessed a high genetic similarity among the genotypes. Like the local comparison, the global comparison of sub-pop I, II and III displayed a marginal similarity among the genotypes of the different sub-pops. But, the individuals in sub-pop III revealed a strong genetic relatedness where all individuals carrying the minor A/A allele accounted for higher trait performance (Supplementary Figure S14A). Equally to the local genetic similitude among genotypes within each sub-pop and among sub-pops, the global comparison revealed a high genetic similarity among individuals within sub-pop III but low genetic relatedness among genotypes of other sub-pops and among the other sub-pops (Supplementary Figure S14B).

#### Root Length (Rl)

We identified two putative QTL located on chromosomes 5H and 7H. According to the relative (RP) performance, the strongest QTL was detected (QRl.7H) on chromosome 7H at 3.82 cM, where the homozygous major allele C/C revealed the highest effect on the phenotype (RP: 7.57%). Genotypes carrying the homozygous minor allele were mostly wild accession from the Middle East. Second QTL effect was located on chromosome 5H between 91.16 and 93.40 cM. This QTL QRl.5H affects the RP by about 5.8% (**Table 1**).

#### Shoot Dry Weight

**FIGURE 2:**
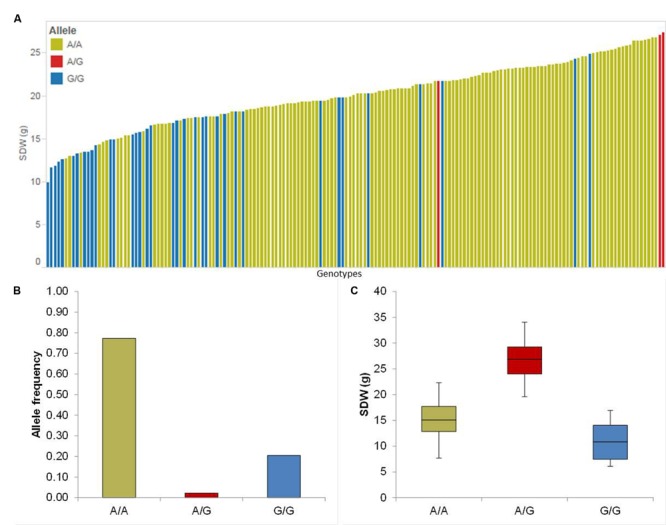
**Quantification of allele based trait effect of QSdw.2H.b. (A)** Pin plot analysis based on allelic effects for Sdw across the whole population. Genotypes are ordered based on their Sdw in 2014 and 2015. **(B)** Allele frequency at QSdw.2H.b. **(C)** Whisker plot for ten randomly selected genotypes per allele to quantify the trait effect of the particular allele, except heterozygous allele. Yellow: Major allele; Red: Heterozygous allele; Blue: Minor allele.

The association mapping for Sdw revealed three significant QTL on chromosomes 2H and 4H (**Table 1**). Chromosome 2H carries the strongest QTL (QSdw.2H.a) between 54.32 and 62.46 cM which affects the RP by 67.65% (**Table 1**). To see the allele-wise differences of the phenotype among the whole population we visualized the strongest QTL effect in a pin plot analysis. Genotypes carrying the heterozygous allele A/G of QSdw.2H.a exhibited the maximum phenotypic effect compared to other allelic variants. By contrast, genotypes bearing the homozygous G/G allele possessed the moderate phenotypic effect (**Figure 2A**). The individual genotypes on x-axis of this pin plot can be identified in Supplementary Table S4. Hereupon, the analysis of the allele-wise distribution for QSdw.2H.a displayed homozygous A/A as major allele and homozygous G/G allele as minor allele (**Figure 2B**). Genotypes featuring heterozygous A/G allele showed the strongest phenotype (average 27 g) while, homozygous minor allele G/G revealed moderate phenotype (average 10 g) (**Figure 2C**).

We compared the genetic relatedness of haplotypic groups to see the genetic background at the local and global genomic level of genotypes bearing homozygous A/A allele and G/G allele. For the local comparison the region between 53.26 and 63.54 cM on chromosome 2H was chosen. The local overall genetic relatedness of sub-pop I and sub-pop II revealed to be distinct due to a high genetic diversity. Similarly, the local comparison of sub-pop I and III and sub-pop II and III showed marginal genetic similarities. Nevertheless, the comparison of genotypes within sub-pop I revealed a high genetic similarity among those genotypes. By contrast, individuals within sub-pop II and III exhibited a high genetic diversity compared to individuals in sub-pop I (Supplementary Figure S15A). Likewise to the local genetic similarity among genotypes within each sub-pop and the genetic similarity among sub-pops, the global comparison revealed a high genetic similarity among genotypes within sub-pop I but low genetic similarities among genotypes of other sub-pops (Supplementary Figure S15B).

#### Tiller Number

**FIGURE 3:**
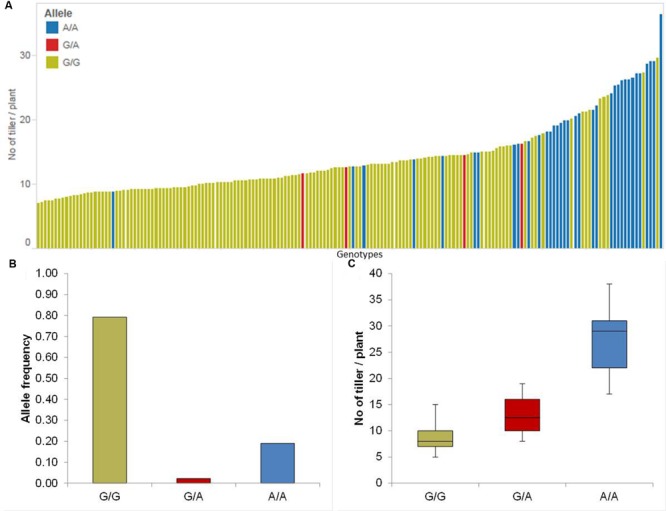
**Quantification of allele based trait effect of QTil.1H. (A)** Pin plot analysis based on allelic effects for Til across the whole population. Genotypes are ordered based on their average tiller number (Til) per plant in 2014 and 2015. **(B)** Allele frequency at QTil.1H. **(C)** Whisker plot for ten randomly selected genotypes per allele to quantify the trait effect of the particular allele, except the heterozygous allele. Yellow: Major allele; Red: Heterozygous allele; Blue: Minor allele.

We identified four significant QTL on chromosomes 1H, 2H and 7H (**Table 1**). The strongest QTL (QTil.1H) was on chromosome 1H between 118.34 and 127.09 cM where the minor allele increased the RP by 50%. The allele-wise differences of the phenotype of all genotypes among the whole population for the most promising QTL (QTil.1H) were visualized in a pin plot diagram. Genotypes bearing the homozygous A/A allele revealed the highest phenotypic effect compared to homozygous G/G allele. While, genotypes possessing the homozygous G/G allele showed marginal phenotypes (**Figure 3A**). The individual genotypes on x-axis of this pin plot can be identified in Supplementary Table S4. The homozygous major allele G/G and homozygous minor allele A/A was revealed by an analysis of the allele-wise distribution. Genotypes carrying the minor allele are mostly wild barley accessions (**Figure 3B**). The strongest QTL effect with an average of 28 tillers per plant was shown by genotypes bearing the homozygous minor allele A/A. On the other hand, genotypes possessing the homozygous major allele G/G exhibited the lowest phenotypic effect (average 8 tillers per plant) (**Figure 3C**).

To analyze the genetic background of genotypes carrying homozygous G/G allele and A/A allele we computed the local and global comparison of genomic groups. The local comparison was performed for the genomic region of QTil.1H between 117.49 and 127.06 cM. The local comparison of sub-pop I and sub-pop II displayed a marginal genetic similarity between these sub-pops. Furthermore, sub-pop I and sub-pop III and sub-pop II and sub-pop III revealed a moderate genetic similarity after comparing their local genetic composition. Moreover, the local comparison of individuals of sub-pop I just showed a low genetic similarity among those genotypes. Additionally, genotypes of sub-pop II exhibited negligible similarity among each other. Contrary, the genotypes within sub-pop III revealed a high genetic similarity to each other but a low genetic similarity to genotypes from other sub-pops (Supplementary Figure S16A). Moreover, the individuals in sub-pop III are carrying the homozygous minor allele A/A exhibiting the highest trait performance. The global comparison of haplotypic groups at genome level revealed a high genetic similarity among genotypes within sub-pop III but low genetic similarities among genotypes of other sub-pops and among other sub-pops, likewise the local comparison (Supplementary Figure S16B).

#### Root-Shoot Ratio (RS)

**FIGURE 4:**
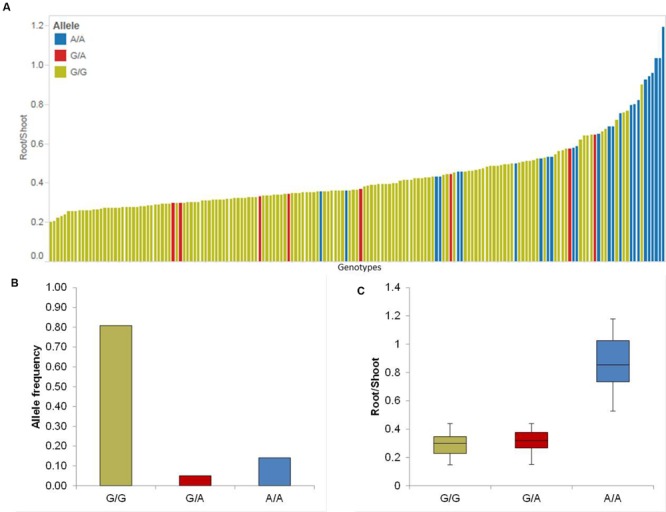
**Quantification of allele based trait effect of QRS.5H. (A)** Pin plot analysis based on allelic effects for RS across the whole population. Genotypes are ordered based on their average RS ratio in 2014 and 2015. **(B)** Allele frequency of QRS.5H. **(C)** Whisker plot for ten randomly selected genotypes per allele to quantify the trait effect of the particular allele, except the heterozygous allele. Yellow: Major allele; Red: Heterozygous allele; Blue: Minor allele.

Five putative QTL were detected on chromosomes 2H, 3H, 4H, 5H, and 7H (**Table 1**). The strongest QTL (QRS.5H) lays on chromosome 5H in the region between 93.40 and 95.00 cM, where the effect of QRS.5H minor allele A/A increases the RP up to 57.14%. To analyze the most promising QTL (QRS.5H), we visualized the allele-wise differences of the phenotype among the whole population in a pin plot (**Figure 4A**). Genotypes which are carrying the homozygous A/A allele featured the strongest phenotypic effect, while genotypes possessing the homozygous G/G allele revealed the lowest phenotype (**Figure 4A**). The individual genotypes on x-axis of this pin plot can be identified in Supplementary Table S4. The analysis of allele-wise distribution of homozygous G/G allele revealed the homozygous G/G allele as major allele and the homozygous A/A allele as minor allele (**Figure 4B**). The homozygous minor allele bearing genotypes showed the strongest phenotype (average 0.9). By contrast, genotypes possessing homozygous major allele G/G exhibited moderate phenotypic effects (average 0.3) (**Figure 4C**).

We analyzed the genetic background of genotypes carrying the homozygous major (G/G) allele and homozygous minor allele (A/A) by comparing local and global haplotypic groups at genome level. The local genetic comparison of QRS.5H was done at a region of 90.18 to 98.89 cM and revealed low genetic similarities among sub-pop I, sub-pop II and sub-pop III. On the other hand, the local comparison of individuals within sub-pop III showed a high genetic similarity among genotypes, except BCC776. While, comparing genotypes within sub-pop II revealed a low genetic similitude among those genotypes. Equally, genotypes of sub-pop I possessed a moderate genetic similarity to each other, compared to genotypes within sub-pop III (Supplementary Figure S17A). The global comparison of selected haplotypic groups displayed a high overall genetic diversity between sub-pop I, II, and III, likewise local comparison of haplotypic groups. The global comparison among genotypes within haplotypic groups revealed a high genetic similarity among individuals of sub-pop III also seen for the local comparison of genotypes in sub-pop III. On the other hand, individuals of sub-pop I and sub-pop II showed a low genetic similarity among each other compared to genotypes within sub-pop III (Supplementary Figure S17B).

### Candidate Gene Analysis

**FIGURE 5:**
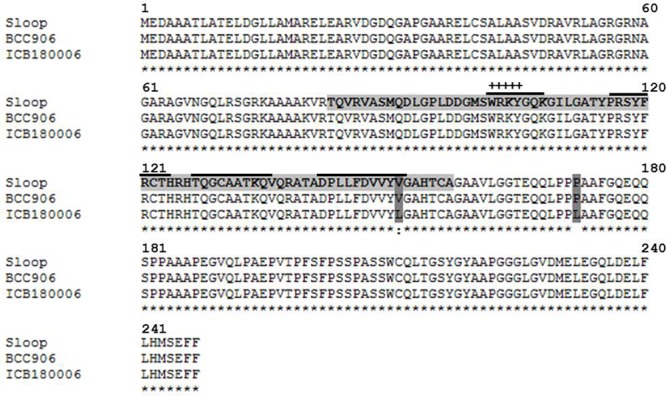
**Protein alignment of WRKY29 transcription factor in cultivated barley Sloop (DQ863113, reference sequence) as well as Morex (BCC 906) and wild barley ICB180006 was made using MAFFT alignment software.** The DNA-binding WRKY domain is indicated by a light gray tag. Amino acid exchanges are indicated by a dark gray tag. “+” indicates the WRKY signature motif. The solid over line indicates an anti-parallel beta-sheet. “^∗^” indicates the identical amino acids in all sequences. “:” indicates conserved substitutions. “ ” indicates non-conserved substitutions.

Putative QTL effects were localized on barley genetic and physical maps to uncover the underlying candidate genes. For this, we focused a hot spot QTL region on chromosome 1H (122.17 cM) associated commonly with shoot and root variation which accounted the highest LOD score for Til. *In silico* analysis of the associated marker BOPA1_7381_1292 with barley Genome Zipper found an essential WRKY transcription factor (WRKY29) gene known for its role in the development of shoot and root (Bakshi and Oelmüller, 2014). Hence, we made full length sequencing of WRKY29 gene in selected genotypes having minor and major QTL alleles for QRdw.1H and QTil.1H. Sequence comparison of selected genotypes along with the reference genotypes revealed two important SNP at positions (+451) and (+515) from ATG (Supplementary Figure S18). The first SNP caused an amino acid substitution of valine 51 (V) to leucine 51 (L) in the conserved domain of WRKY29 protein. The second mutation resulted in the substitution of proline 72 (P) to leucine 72 (L) at the position next to conserved domain (**Figure 5**).

**FIGURE 6:**
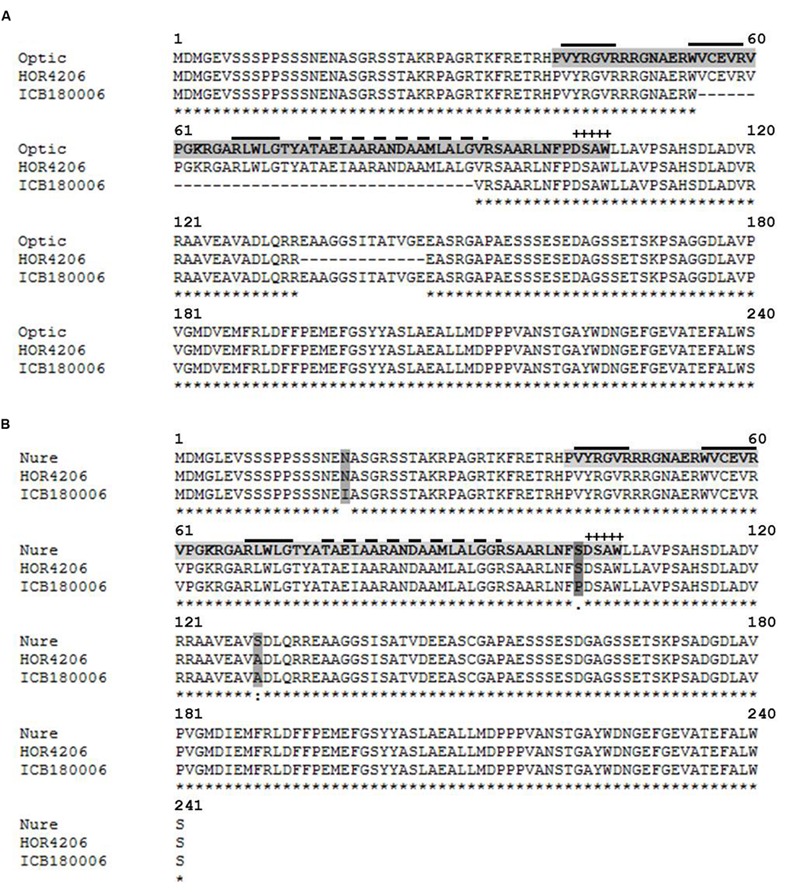
**Protein alignment of transcription factors CBF10B and CBF10A in different barley accessions using MAFFT alignment.** The DNA (CRT/DRE) binding AP2/ERF domain is indicated by a light gray tag. The dark gray tag indicates amino acid exchanges. “+” indicates the CBF signature motif DSAW signature motif (Jaglo et al., 2001). The solid over line indicates an anti-parallel beta-sheet (Allen et al., 1998). The dashed over line indicates an amphipathic alpha-helix. “^∗^” indicates the identical amino acids in all sequences. “:” indicates conserved substitutions. “.” indicates semi-conserved substitutions. “ :” indicates non-conserved substitutions. **(A)** Alignment of CBF10B in cultivated barley Optic (AAX28956, reference sequence) and Cape (HOR 4206) as well as wild barley ICB180006. **(B)** Alignment of CBF10A cultivated barley Nure (DQ445241, reference sequence) and Cape (HOR4206) as well as wild barley ICB180006.

The second candidate region harbors a major QTL effect (QRdw.5H) that accounted for the highest genetic variance for Rdw and was found to be drought inducible as it showed significant M and M × T interaction effects simultaneously. We found drought related regulatory genes CBF10B/CBF10A around 5089 bp away from associated marker BOPA2_12_30850. Sequence analysis of CBF10B among selected genotypes having major and minor QTL alleles of QRdw.5H revealed a major deletion 111 bp at position +162 (Supplementary Figure S19). This mutation resulted in 37 amino acid deletion in the conserved domain of CBF10B allele originating from wild accession ICB180006 (**Figure 6A**). Sequence analysis of CBF10A in the similar genotypes resulted in seven SNP at positions +53, +168, +177, +219, +252, +294, +304 from ATG (Supplementary Figure S20). These SNP resulted in amino acid substitutions of which the change of T to C at position +304 caused a substitution of serine (S) to proline (P) in the conserved domain of CBF10A gene between major and minor QTL alleles (**Figure 6B**).

## Discussion

Genetic diversity of barley natural population is known for its inherent morphological novelties, geographic and environmental adaptations. These features enable barley genotypes to grow from boreal to equatorial regions world-wide. Overall, this trait diversity is the product of plant evolution and related forces like natural selection. The first objective of the present work was to establish a state of the art genetic resources based on morphological novelties, geographic distribution and inherent environmental adaptation. Secondly, we employed genome-wide association approach using a dense genetic map to dissect the genetic basis of root and shoot traits as well as their putative role in drought adaptation. For this, we focused primarily the root trait variation, to find major genetic players contributing to different root systems in barley and secondly to dissect the putative genetic interplay of root and shoot traits. It has been reported that the root architecture takes major role in plant adaptation to drought (Chloupek et al., 2010; Wasson et al., 2012; Barati et al., 2015). Although, numerous GWAS studies have been made on barley diversity analysis by Nandha and Singh (2014) and Russell et al. (2014), but genetic dissection of root traits remained fragmented due to difficulty for its phenotypic evaluations. To our knowledge, the current work presents the first study of its kind that utilized world-wide germplasm of barley to investigate the essential root and related shoot trait variations using a high resolution SNP map through GWAS.

Phenotypic evaluation showed significant variations for Rdw, Sdw, Til, and RS under control and drought conditions between various genotypes indicating a broad genetic and phenotypic variance within the global barley population. Particularly, wild barley accessions showed higher values for Rdw, Til, and RS as compared to cultivated varieties. Nandha and Singh (2014) studied 27 wild accessions originating from the Middle East as well as 20 cultivars and found the presence of vital exotic alleles in determining root trait variation. Tyagi et al. (2011) reported significant environmental adaptation among the wild accessions from the Fertile Crescent. These present data also showed high correlation of Rdw and Til indicating the presence of common genetic components influencing root and shoot traits. These results are in line with Anderson-Taylor and Marshall (1983) as well as Narayanan and Prasad (2014), who also found close relationship of root traits and Til per plant in barley and other crops. Although, phenotypic evaluations were made across the years 2014 and 2015 but we found significant heritability of most of the root and shoot traits except Rl suggesting the genetic control of these traits (Supplementary Table S2). Heritability is the most important criteria for selecting traits in plant breeding and hence, traits possessing higher heritability across different environments could be prime leads for breeding.

The present GWAS detected 17 QTL for five root and shoot traits. The number of QTL was relatively low because we employed a highly stringent criteria of backward forward selection of significant SNP markers using higher threshold of probability and FDR (Miyagawa et al., 2008). A major reason of this strict statistical threshold was to get rid of the false positive QTL effect. Among the detected QTL at 14 (78%) loci the preeminence of exotic alleles from the wild barley accessions was associated with increase in trait values. Likewise, at 7 (39%) loci the exotic alleles showed significant interaction with drought treatment. These data thus indicated the presence of valuable alleles in the exotic germplasm for the improvement of RS attributes and drought stress tolerance. Quantification of these QTL alleles is always a challenge in association panels due to their heterogeneous background. Therefore, we made a pin plot analysis of the most promising QTL to visualize distribution of trait values population wide. Later, we selected extreme groups of the homozygous major and minor alleles for the quantification of allelic effects on a given trait. In order to confirm the haplotype relationship of genotypes contributing to individual QTL effect, we selected 30 genotypes randomly for each QTL effect and analyzed their genetic relatedness at local and global genome levels. This analysis showed that the wild accessions contributing to a given QTL effect revealed higher genetic similarities at both local and global genome levels. Zhao et al. analyzed genotype relatedness by calculating the identity by state (IBS) in GWAS analysis for QTL quantification to explain phenotypic variations among genotypes of a rice association panel (Zhao et al., 2011). They also detected phenotypic similarities among genotypes from same geographical locations.

The strongest QTL detected in the present study was localized on chromosome 1H (122.17 cM) where a unique exotic allele influenced root and shoot variation. The highest LOD score (102.61) at QTil.1H indicated the role of a major gene controlling Til. Similar marker (BOPA1_7381-1292) showed significant association with QTL QRdw.1H but at relatively lower LOD score (11.57). These data suggested that this locus may underlie a major gene that controls primarily the Til. However, excessive tillering resulted in the initiation of more nodal roots suggested the dependence of shoot and root development. Similar results were reported earlier by Arifuzzaman et al. (2014) and Naz et al. (2014) where a putative QTL region was found for Rdw and related shoot traits on chromosome 1H in barley. To find the putative candidate gene underlying this variation, we identified 10 putative genes of different categories in the targeted QTL interval using barley genome sequence (Mayer et al., 2012). Among these, based on the functional relevance and existing literature we suspected the role of a WRKY transcription factor, WRKY29 in this major trait variation (Rushton et al., 2010; Bakshi and Oelmüller, 2014). Due to sequence comparison of the genotypes carrying major and minor QTL alleles, we found a crucial amino acid substitution mutation, from V51 (Valine) to L51 (Leucine) in the conserved WRKY DNA-binding domain (**Figure 5**). Therefore, we suggest this substitution mutation may change DNA-binding affinity among the selected haplotypes. However, further experiments are needed to test its role in a more isogenic background. According to Betts and Russell a substitution to L (Leucine) is crucial for secondary structures because of leucine’s properties (Betts and Russell, 2007). Hydrophobic leucine prefers to bury in hydrophobic protein cores and being in alpha-helices in contrast to valine which prefers to be in beta-sheets. Therefore, it seems possible that the exchange from V51 to L51 leads to a wrongly folded beta-sheet because of the involvement of V51 in the fourth beta-sheet of WRKY DNA-binding domain (Zhu et al., 1993).

The second promising QTL was identified on chromosome 5H that showed marker main as well as marker x treatment effects indicating the role of an exotic QTL allele in root system variation under control and drought stress conditions. There are a lot of reports that advocate the patterning of root under stress conditions (Chloupek et al., 2010; Naz et al., 2012; Narayanan et al., 2014). To find genetic component behind this novel adaptation under drought, we searched candidate genes in the targeted QTL region using Genome Zipper of barley (Mayer et al., 2012). We found altogether 12 putative candidate genes of which only two were related (C-repeat binding factor, CBF10B/CBF10A) transcription factor having a regulatory function under drought conditions. The function of CBF transcription factors in drought stress tolerance has been reported in many cases (Akhtar et al., 2012; Nakashima et al., 2014). Notably, both genes CBF10B/CBF10A and associated SNP marker were lying on the same genomic contig on the physical map. Therefore, we sequenced both genes in selected genotypes harboring major and minor QTL alleles for QRdw.5H. Sequencing comparison of full length CBF10B gene among the selected genotypes revealed a macro mutation in term of large deletion of 37 amino acids of the conserved domain in the wild barley accession as compared to cultivated genotypes (**Figure 6A**). Whereas, we found a vital amino acid substitution from S102 (Serine) to P102 (Proline) within the AP2/ERF DNA-binding domain (**Figure 6B**). The shift of serine to proline was suggested as crucial by Betts and Russell because of structural properties of proline. Although, there exists qualitative gene polymorphism among barley genotypes, we hypothesize there may be a complex and redundant regulation of this gene in root patterning under control and drought stress conditions (Betts and Russell, 2007). Previously, Naz et al. (2012) mapped a large QTL region for root system variation using introgression line on the long arm of chromosome 5H which putatively underlie Vrn-H1 locus. However, the above mentioned QTL effect does not correspond to Vrn-H1 region suggesting the novelty of this putative QTL allele in root system determination under drought stress conditions.

The present GWAS analyses also identified a major QTL QSdw.2H.a for Sdw that explained the highest genetic variance (33.7%) on chromosome 2H (58.99 cM). Notably, this QTL effect appeared as prominent heterotic effect where the heterozygous alleles resulted in a major increase in Sdw as compared to homozygous alleles. This QTL region seems to underlie major circadian clock gene Ppd-H1 that controls plant development and early heading in barley under long day conditions. Early and delayed heading are usually correlated with lesser and more Sdw, respectively (Wang et al., 2010; Arifuzzaman et al., 2014). A dominant early heading allele Ppd-H1 has been reported in wild barley accession ISR42-8 and its effect has been confirmed in introgression lines S42IL-107 harboring ISR42-8 Ppd-H1 allele in Scarlett (spring type) background (Schmalenbach et al., 2009; Arifuzzaman et al., 2014). However, here we identified two unique haplotypes HOR2692 (Iranian wild accession) and NGB4673 (Landrace from Afghanistan) having heterozygous alleles at QTL QSdw.2H.b. The heterotic effect of this QTL on enhanced Sdw weight led us to surmise that these genotypes may underlie new variants of Ppd-H1. These data may also indicate new dimension of Ppd-H1 regulation in term of heterosis in barley.

Taken together, the present GWAS has successfully screened natural diversity of barley to identify novel variants for root and shoot attributes that seems beneficial for improving the inferior rooting system of cultivated varieties. Further, the genetic determination of these phenotypes revealed important QTL/candidate genes which provide an opportunity for further research to characterize the role of these genes more precisely and to understand the genetic mechanisms of barley root and shoot development across diverse climatic and geographic conditions.

## Materials and Methods

### Ethics Statement

The present study employed quantitative genetics approach to identify genome-wide QTL controlling fibrous root system and related shoot variation which can be useful to improve root attributes for water use efficiency and drought stress tolerance in barley.

### Plant Material

The studied germplasm panel contains 179 different genotypes that were collected in 38 countries across the globe (Supplementary Table S1). It includes 48 *Hordeum vulgare* ssp. *spontaneum* (wild) accessions and 131 *Hordeum vulgare* L. ssp. *vulgare* (cultivar) accessions. The latter is made up of 72 landraces and 59 modern cultivars. The seeds were provided by Leibniz Institute for Plant Genetic and Crop Science (IPK, Gatersleben, Germany), Nordgen (NGB, Alnarp, Sweden), ICARDA (Beirut, Lebanon).

### Genotyping

The germplasm panel was genotyped using the Illumina 9K iSelect SNP chip and the analysis was performed at TraitGenetics (TraitGenetics GmbH, Seeland OT Gatersleben, Germany) (Mayer et al., 2012). The 7842 obtained markers were processed using the criteria as described by Miyagawa et al. (Miyagawa et al., 2008): minor allele frequency (MAF) >0.05; <0.95 for SNP call rate, removing the monomorphic ones and performed using SAS 9.3 (SAS Institute 2008, Cary, NC, USA). A total of 5892 polymorphic markers fulfilled the mentioned cleaning criteria and were used for further analysis. The marker positions for the high density map according to Comadran et al. (2012).

### Population Structure Analysis

A population structure analysis was performed with 5892 SNP marker using the software package STRUCTURE v2.3.4 with a Bayesian Markov Chain Monte Carlo (MCMC) approach. Settings of calculation according to Morrell and Clegg (2007): Default admixture and independent allele frequency models were adapted; *K* was set from 1 to 20; burnin period was set to 100000 and the number of MCMC replications after each burnin to 300000. The iteration number was 10. Detection of the value of ΔK was performed with a Markov clustering algorithm implemented in CLUMPAK (Kopelman et al., 2015).

The Kinship matrix was calculated with rrBLUP. FactoMineR was used to calculate the principal component analysis (PCA). The LD for the whole population and groups of genotypes with the same biological status (sub-pop 1 = cultivars, sub-pop 2 = landraces, sub-pop 3 = wild barley) was performed with 5892 polymorphic SNP marker. The PCA, Kinship matrix and the LD were created by using the statistical software R, respectively.

The analysis of the genetic distance of randomly selected genotypes was determined by calculating the Rogers distance (PROC distance) using the software package SAS 9.3. The genetic relationship of those selected genotypes was compared locally and globally. For the local comparison a 5 cM area left and right of the significant marker was chosen and the Rogers distance was calculated for all markers within this 10 cM region. For the global comparison, the Rogers distance was computed for all 5892 polymorphic SNP marker.

### Phenotypic Evaluation of Root and Shoot Related Traits

Phenotypic evaluation for selected genotypes was carried out in years 2014 and 2015. In each year the individuals were replicated four times and arranged in a split plot design with two treatments (control and drought) in sub-plots. The sub-plots were separated in lines in which they were arranged randomly in a foil tunnel. One seed of individual accession was sown in plastic pots (19.5 cm × 25.5 cm) containing a mixture of topsoil (40%) and natural sand (60%) (Cordel & Sohn, Salm, Germany). A drip water irrigation system (Netafilm, Adelaide, Australia) was installed to water the pots three times a day. To determine the volumetric moisture content (VMC) the DL2e Data Logger soil moisture sensor was used. At plant development stage BBCH 31–34 (Lancashire et al., 1991) the water supply was reduced until reaching the VMC of 5% within 2 weeks. The soil moisture was kept at 5% for another two weeks to conduct the drought stress treatment. Control plants were irrigated without interruption.

Five root and shoot related traits were evaluated as followed: The shoots were cut off from roots 0.5 cm above the RS junction. Afterward, roots were washed manually and traits were evaluated. Rdw: Roots were dried in a drying chamber at 50°C for 7 days and weighed in grams (g), thereupon. Rl: The length of each root was measured from the stem base to the root tip by spreading the root on a measuring tape (cm). Sdw: Shoots were dried at 50°C in a drying chamber for 7 days and weighed in grams (g), thereupon. Til per plant: Before sampling, the total c was counted for each plant. RS ratio: Dividing the Rdw by Sdw.

### Statistical Analysis

A summary statistic was performed by using the software package SAS 9.3. The analysis of variance (ANOVA) was computed with the general linear model (PROC GLM) procedure:

(1) $$Y_{ijk}=\mu +T_{i}+R_{j}\left(T_{i}\right)+G_{k}+G_{k}\;x\;T_{i}+G_{k}\;x\;Y_{l}+G_{k}\;x\;T_{i}\;x\;Y_{l}+\varepsilon_{ijk}$$

where μ is the general mean, *T_i_* the fixed effect of the *i*-th treatment, *R_j_(T)* the random effect of the *i*-th treatment between the *j*-th replication, *G_k_* the fixed effect of the *k*-th genotype, *G_K_xT_i_* the fixed interaction effect of the *k*-th genotype with *i*-th treatment, *G_K_xT_l_* is the fixed interaction effect of the *k*-th genotype with *l*-th year and *G_K_xT_i_xY_l_* is the fixed multiple interaction effect of the *k*-th genotype with *i*-th treatment and *l*-th year.

To calculate the coefficients for broad-sense heritability (*H*^2^) (Falconer and Mackay, 1996; Holland et al., 2003) variance components were estimated with PROC VARCOMP procedure in SAS: Variance of genotype (V*_G_*), variance of genotype by treatment (V*_GxT_*), the variance of genotype by year (V*_GxY_*) and the variance of the experimental error (V*_E_*). Respectively, *t, y*, and *r* are the number of treatments (*t* = 2), the number of years (*y* = 2) and the average number of replications (*r* = 3.8):

(2) $$H^{2}=\frac{{\text{V}}_{G}}{{\text{V}}_{G}\;+\;\frac{{\text{V}}_{G\;x\;T}}{t}\;+\;\frac{{\text{V}}_{G\;x\;Y}}{y}\;+\;\frac{{\text{V}}_{E}}{tyr}}$$

A Pearson correlation was performed by using the PROC CORR procedure in SAS. The correlation coefficient was calculated between the five different root and shoot traits: Rdw, Rl, Sdw, Til, and RS, respectively.

### Association Mapping Model

The Association mapping was performed using the mixed linear model in the PROC MIXED procedure in SAS 9.3.

(3) $${\text{Y}}_{ij}\;=\;\mu +\;M_{i}+\;L_{i}\left(M_{i}\right)+\;\varepsilon_{ij}$$

where *Y_ij_* is the phenotypic value; μ is the general mean; *M_i_* is the fixed effect of *i*-th marker genotype/haplotype; *L_j_ (M_i_)* is the random effect of *j*-th barley line nested within *i*-th marker genotype/haplotype and 𝜀*_ij_* is the residual. “Year” was set as a factor for replication. Therefore, “year” is not included in this model itself. To determine traits of interest in the genome-wide detection analysis a log of odds (LOD) threshold with *p-*value ≤0.0001 and 1,000 permutations was determined. The QTL-model comprises an iterative multi-locus procedure. Therefore, the most informative SNP (QTL) was set as a fixed factor during each calculation iteration step. All remaining marker were again incorporated in the next iteration round and reanalyzed. The starting point of next calculation round was determined by the result of the previous iteration. This procedure was repeated until no marker could be detected, which led to a reduction of significant marker and thereby a reduced number of false positive QTLs. A confidence interval of 5 cM was chosen on both sides of the most significant SNP and designated as putative QTL. SNPs were combined to one joint QTL depending on their estimated (significant) *p*-value from the first iteration of the multi-locus procedure. Therefore, the size of the genetic interval was dependent on the significance value of flanking SNPs. A “leave-20%-out” cross validation procedure was used to increase the validity of all significant SNPs. A reduced dataset with randomly excluded twenty percent of the phenotypic information from the original dataset was newly calculated as described above. Thus new calculated mean value, in X times iterated procedure, was set as a new *p*-value to define significant SNPs (Sannemann et al., 2015).

## Author Contributions

AN, JL, and SR conceptualized the present research. SR did phenotyping and genotyping for association mapping. SR, JL, and AN conducted the data analysis. SR, AN, and AK performed the candidate gene analysis. SR and AN wrote the manuscript.

## Conflict of Interest Statement

The authors declare that the research was conducted in the absence of any commercial or financial relationships that could be construed as a potential conflict of interest.
