# Supplementary material for: Genome-Wide Association Mapping in the Global Diversity Set Reveals New QTL Controlling Root System and Related Shoot Variation in Barley
Source: Front Plant Sci. 2016 Jul 19;7:1061. doi: 10.3389/fpls.2016.01061 (PMC4949209; doi:10.3389/fpls.2016.01061)
Supplement: Supplementary file 1 [file Table_1.PDF]

Table S1: . Global barley population. List of all Genotypes which are included in the global barley population with accession number, region and country of collection and the biological status (Bio. status)

No	Accession	Namecode	Scientific Name	Region	Country	Biological status	Altitude	Longitude	Latitude
1	HOR 16097	RBC001	*Hordeum sp.*	Africa	Egypt	Cultivar			
2	HOR 19027	RBC002	*Hordeum sp.*	Africa	Egypt	Landrace			
3	HOR 20117	RBC003	*Hordeum sp.*	Africa	Egypt	Landrace			
4	HOR 19308	RBC004	*Hordeum sp.*	Africa	Egypt	Landrace			
5	BCC 126	RBC005	*Hordeum vulgare* L. *convar. vulgare var. hybernum Viborg*	Africa	Marocco	Landrace			
6	BCC 149	RBC006	*Hordeum vulgare* L. *convar. vulgare var. hybernum Viborg*	Africa	Marocco	Landrace			
7	HOR 13412	RBC007	*Hordeum vulgare* L. *convar. vulgare var. hybernum Viborg*	Africa	Marocco	Landrace	NN 596	31°22'00'' N	008°31'00'' W
8	BCC 131	RBC008	*Hordeum vulgare* L. *convar. vulgare var. hybernum Viborg*	Africa	Marocco	Landrace			
9	HOR 9838	RBC009	*Hordeum spontaneum* K.Koch	Africa	Libya	Wild type	NN 424	27°2'16'' N	14°25'36'' E
10	HOR 9721	RBC010	*Hordeum spontaneum* K.Koch *var. spontaneum*	Africa	Libya	Wild type	NN 590	32°44'54'' N	21°45'38'' E
11	HOR 10164	RBC011	*Hordeum spontaneum* K.Koch *var. ischnatherum (Coss.)* Thell.	Africa	Libya	Wild type	NN 300	32°6'34'' N	21°10'9'' E
12	HOR 9840	RBC012	*Hordeum spontaneum* K.Koch *var. ischnatherum (Coss.)* Thell.	Africa	Libya	Wild type	NN 656	32°47'46'' N	22°7'18'' E
13	HOR 16287	RBC013	*Hordeum sp.*	Africa	Sudan	Landrace			
14	HOR 2589	RBC014	*Hordeum sp.*	Africa	Sudan	Landrace			
15	HOR 16359	RBC015	*Hordeum sp.*	Africa	Sudan	Landrace			
16	HOR 15956	RBC016	*Hordeum sp.*	Africa	Sudan	Landrace			
17	ICB 180006	RBC017	*Hordeum spontaneum* K.Koch	Middle East	Syria	Wild type	NN 250	35°49'42'' N	036°18'28''E
18	ICB 180862	RBC018	*Hordeum spontaneum* K.Koch	Middle East	Syria	Wild type			
19	ICB 180902	RBC019	*Hordeum spontaneum* K.Koch *var. ischnatherum (Coss.)* Thell.	Middle East	Syria	Wild type			
20	IG 121857	RBC020	*Hordeum spontaneum* K.Koch	Middle East	Syria	Wild type	NN 1059	32°33'00''N	036°35'42''E
21	HOR 930	RBC021	*Hordeum vulgare* L. *convar. vulgare var. hybernum Viborg*	Middle East	Turkey	Landrace			
22	HOR 14953	RBC022	*Hordeum sp.*	Middle East	Turkey	Landrace			
23	HOR 14936	RBC023	*Hordeum sp.*	Middle East	Turkey	Landrace			
24	HOR 19883	RBC024	*Hordeum sp.*	Middle East	Turkey	Landrace			
25	ICB 180092	RBC025	*Hordeum spontaneum* K.Koch *var. spontaneum*	Middle East	Palestine	Wild type			
26	ICB 180117	RBC026	*Hordeum spontaneum* K.Koch *var. ischnatherum (Coss.)* Thell.	Middle East	Palestine	Wild type			
27	ICB 180410	RBC027	*Hordeum spontaneum* K.Koch	Middle East	Palestine	Wild type			
28	ICB 180994	RBC028	*Hordeum spontaneum* K.Koch	Middle East	Palestine	Wild type	NN 54	31°40'00''N	034°34'00''E
29	ICB 181160	RBC029	*Hordeum spontaneum* Koch *var. turcomanicum* Vav.	Middle East	Iran	Wild type			
30	HOR 2684	RBC030	*Hordeum spontaneum* K.Koch *var. spontaneum*	Middle East	Iran	Wild type	NN 110	32°6'21'' N	048°50'2'' E
31	HOR 2692	RBC031	*Hordeum spontaneum* K.Koch *var. spontaneum*	Middle East	Iran	Wild type	NN 120	32°23'36'' N	047°38'25'' E
32	HOR 2687	RBC032	*Hordeum spontaneum* K.Koch *var. spontaneum*	Middle East	Iran	Wild type	NN 90	31°35'29'' N	049°5'20'' E
33	ICB 181442	RBC033	*Hordeum spontaneum* K.Koch	Middle East	Jordan	Wild type	NN 782	31°17'51''N	035°50'41''E
34	ICB 181418	RBC034	*Hordeum spontaneum* K.Koch	Middle East	Jordan	Wild type	NN 812	31°46'47''N	035°48'00''E
35	ICB 180013	RBC035	*Hordeum spontaneum* K.Koch	Middle East	Jordan	Wild type	NN 480	32°14'25''N	035°51'55''E

36	ICB 181268	RBC036	*Hordeum spontaneum* K.Koch	Middle East	Jordan	Wild type	NN750	32°18'6''N	035°55'17''E
37	ICB 180007	RBC037	*Hordeum spontaneum* K.Koch	Middle East	Jordan	Wild type	NN 591	32°29'15''N	035°55'39''E
38	ICB 180260	RBC038	*Hordeum spontaneum* K.Koch *var. spontaneum*	Middle East	Israel	Wild type	NN 36	33°00'00''N	035°08'00''E
39	HOR 9470	RBC039	*Hordeum spontaneum* K.Koch *var. spontaneum*	Middle East	Israel	Wild type			
40	ICB 180329	RBC040	*Hordeum sp.*	Middle East	Israel	Wild type	NN 83	31°26'00'' N	34°29'00'' E
41	ICB 180508	RBC041	*Hordeum sp.*	Middle East	Israel	Wild type			
42	HOR 20921	RBC042	*Hordeum sp.*	Middle East	Israel	Landrace			
43	ICB 180046	RBC043	*Hordeum spontaneum* K.Koch	Middle East	Iraq	Wild type	NN 323	36°00'00''N	043°31'00''E
44	ICB 180069	RBC044	*Hordeum spontaneum* K.Koch	Middle East	Iraq	Wild type	NN 470	34°48'00''N	045°36'00''E
45	HOR 11106	RBC045	*Hordeum sp.*	Middle East	Iraq	Wild type			
46	HOR 2514	RBC046	*Hordeum spontaneum* K.Koch *var. transcaspicum Vavilov*	Asia	India	Wild type			
47	HOR 11421	RBC047	*Hordeum sp.*	Asia	India	Landrace	NN 2880	31°41'31'' N	77°31'35'' E
48	HOR 8367	RBC048	*Hordeum vulgare* L. *convar. vulgare var. himalayense* (Rittig) Körn.	Asia	India	Landrace			
49	HOR 8372	RBC049	*Hordeum vulgare* L. *convar. vulgare var. hybernum Viborg*	Asia	India	Landrace			
50	HOR 7603	RBC050	*Hordeum vulgare* L. *convar. vulgare var. himalayense* (Rittig) Körn.	Asia	Pakistan	Landrace	NN 2830	36°05'40'' N	074°04'35'' E
51	HOR 18401	RBC051	*Hordeum spontaneum* K.Koch	Asia	Pakistan	Wild type			
52	HOR 7599	RBC052	*Hordeum vulgare* L. *convar. vulgare var. himalayense* (Rittig) Körn.	Asia	Pakistan	Landrace	NN 2100	36°17'40'' N	073°46'57'' E
53	ICB 181243	RBC053	*Hordeum spontaneum* K.Koch	Asia	Pakistan	Wild type	NN 1560	30°18'00'' N	066°54'00'' E
54	BCC 732	RBC054	*Hordeum vulgare* L. *convar. vulgare var. hybernum Viborg*	Asia	Nepal	Landrace			
55	BCC 776	RBC055	*Hordeum vulgare* L. *convar. vulgare var. breviaristatum*	Asia	Nepal	Landrace			
56	HOR 17616	RBC056	*Hordeum sp.*	Asia	Nepal	Landrace			
57	HOR 18945	RBC057	*Hordeum sp.*	Asia	Nepal	Landrace			
58	HOR 16714	RBC058	*Hordeum sp.*	Asia	China	Landrace			
59	HOR 1479	RBC059	*Hordeum vulgare* L. *convar. vulgare* (Vavilov & Orlov) Mansf.	Asia	China	Landrace	NN 3685	29°21'00'' N	090°39'00'' E
60	HOR 1510	RBC060	*Hordeum vulgare* L. *convar. vulgare var. revelatum* Körn.	Asia	China	Landrace	NN 3650	29°38'59'' N	091°05'59'' E
61	HOR 1566	RBC061	*Hordeum vulgare* L. *convar. vulgare var. tibetanum* (Vavilov & Orlov) Mansf.	Asia	China	Landrace	NN 4076	29°15'19'' N	090°49'59'' E
62	HOR 56	RBC062	*Hordeum vulgare* L. *convar. vulgare var. hybernum Viborg*	Asia	China	Landrace			
63	NGB4668	RBC063	*Hordeum vulgare ssp. vulgare*	Asia	Afghanistan	Landrace			
64	NGB9606	RBC064	*Hordeum vulgare ssp. vulgare*	Asia	Afghanistan	Landrace			
65	NGB4673	RBC065	*Hordeum vulgare ssp. vulgare*	Asia	Afghanistan	Landrace			
66	NGB6952	RBC066	*Hordeum vulgare ssp. vulgare*	Asia	Afghanistan	Landrace			
67	NGB9599	RBC067	*Hordeum vulgare ssp. vulgare*	Asia	Afghanistan	Landrace			
68	NGB8872	RBC068	*Hordeum vulgare ssp. vulgare*	Asia	Afghanistan	Landrace			
69	ICB 181498	RBC069	*Hordeum spontaneum* K.Koch	Asia	Uzbekistan	Wild type	NN 350	41°9'58''N	069°02'00''E
70	IG 124000	RBC070	*Hordeum spontaneum* K.Koch	Asia	Uzbekistan	Wild type	NN 1450	39°42'00''N	068°02'45''E
71	IG 124017	RBC071	*Hordeum spontaneum* K.Koch	Asia	Uzbekistan	Wild type	NN 700	40°00'00''N	067°05'15''E

72	BCC 282	RBC072	*Hordeum vulgare* L.	Asia	Uzbekistan	Landrace			
73	ICB 180211	RBC073	*Hordeum spontaneum* K.Koch	Asia	Turkmenistan	Wild type	NN 1530	37°42'59'' N	058°24'50'' E
74	ICB 180215	RBC074	*Hordeum spontaneum* K.Koch	Asia	Turkmenistan	Wild type			
75	ICB 180217	RBC075	*Hordeum spontaneum* K.Koch	Asia	Turkmenistan	Wild type	NN 250	37°40'00''N	065°35'00'' E
76	ICB 181492	RBC076	*Hordeum spontaneum* Koch *var. transcaspicum* Vavilov	Asia	Turkmenistan	Wild type	NN 456	38°02'00''N	058°00'00''E
77	HOR 18647	RBC077	*Hordeum sp.*	Asia	Japan	Landrace			
78	HOR 15779	RBC078	*Hordeum sp.*	Asia	Japan	Landrace			
79	BCC 613	RBC079	*Hordeum vulgare* L.	Asia	Japan	Cultivar			
80	HOR 19848	RBC080	*Hordeum sp.*	Asia	Japan	Landrace			
81	BCC 891	RBC081	*Hordeum vulgare* L.	North America	USA	Cultivar			
82	BCC 906	RBC082	*Hordeum vulgare* L.	North America	USA	Cultivar			
83	BCC 817	RBC083	*Hordeum vulgare* L.	North America	USA	Cultivar			
84	BCC 875	RBC084	*Hordeum vulgare* L.	North America	USA	Cultivar			
85	BCC 801	RBC085	*Hordeum vulgare* L.	North America	Canada	Cultivar			
86	BCC 852	RBC086	*Hordeum vulgare* L. *convar. vulgare var. hybernum Viborg*	North America	Canada	Cultivar			
87	BCC 881	RBC087	*Hordeum vulgare* L.	North America	Canada	Cultivar			
88	BCC 888	RBC088	*Hordeum vulgare* L.	North America	Canada	Cultivar			
89	HOR 4124	RBC089	*Hordeum vulgare* L. *convar. vulgare var. hybernum Viborg*	South America	Mexico	Landrace			
90	HOR 13597	RBC090	*Hordeum sp.*	South America	Mexico	Wild type			
91	BCC 848	RBC091	*Hordeum vulgare* L. *convar. vulgare var. hybernum Viborg*	South America	Mexico	Cultivar			
92	BCC 900	RBC092	*Hordeum vulgare* L.	South America	Mexico	Cultivar			
93	HOR 7443	RBC093	*Hordeum vulgare* L. *convar. vulgare var. hybernum Viborg*	South America	Bolivia	Landrace			
94	HOR 7446	RBC094	*Hordeum vulgare* L. *convar. vulgare var. hybernum Viborg*	South America	Bolivia	Landrace			
95	BCC 882	RBC095	*Hordeum vulgare* L.	South America	Bolivia	Cultivar			
96	BCC 928	RBC096	*Hordeum vulgare* L.	South America	Bolivia	Cultivar			
97	HOR 2981	RBC097	*Hordeum vulgare* L.	South America	Chile	Cultivar			
98	BCC 871	RBC098	*Hordeum vulgare* L.	South America	Chile	Cultivar			
99	HOR 20110	RBC099	*Hordeum sp.*	South America	Chile	Landrace			
100	HOR 14485	RBC100	*Hordeum sp.*	South America	Chile	Landrace			
101	BCC 844	RBC101	*Hordeum vulgare* L. *convar. vulgare var. hybernum Viborg*	South America	Colombia	Cultivar			
102	BCC 921	RBC102	*Hordeum vulgare* L. *convar. vulgare var. hybernum Viborg*	South America	Colombia	Cultivar			
103	HOR 10843	RBC103	*Hordeum vulgare* L. *convar. vulgare var. hybernum Viborg*	South	Colombia	Landrace	NN 2000	5°45'15'' N	73°34'37'' W

				America					
104	HOR 10845	RBC104	*Hordeum vulgare* L. *convar. vulgare var. hybernum Viborg*	South America	Colombia	Landrace	NN 2790	1°12'59'' N	77°23'30'' W
105	BCC 927	RBC105	*Hordeum vulgare* L. *convar. vulgare var. hybernum Viborg*	South America	Peru	Cultivar			
106	HOR 19577	RBC106	*Hordeum sp.*	South America	Peru	Landrace			
107	HOR 7449	RBC107	*Hordeum vulgare* L. *convar. vulgare var. hybernum Viborg*	South America	Peru	Landrace			
108	HOR 9565	RBC108	*Hordeum vulgare* L. *convar. vulgare var. hybernum Viborg*	South America	Peru	Landrace			
109	HOR 16345	RBC109	*Hordeum sp.*	South America	Uruguay	Landrace			
110	HOR 17307	RBC110	*Hordeum sp.*	South America	Uruguay	Landrace			
111	BCC 862	RBC111	*Hordeum vulgare* L.	South America	Uruguay	Cultivar			
112	BCC 896	RBC112	*Hordeum vulgare* L.	South America	Uruguay	Cultivar			
113	HOR 35	RBC113	*Hordeum vulgare* L. *convar. vulgare var. hybernum Viborg*	Australia	Australia	Cultivar			
114	HOR 4206	RBC114	*Hordeum vulgare* L. *convar. vulgare var. hybernum Viborg*	Australia	Australia	Cultivar			
115	HOR 4278	RBC115	*Hordeum vulgare* L. *convar. vulgare var. hybernum Viborg*	Australia	Australia	Cultivar			
116	HOR 18209	RBC116	*Hordeum sp.*	Australia	Australia	Cultivar			
117	HOR 20173	RBC117	*Hordeum sp.*	Australia	Australia	Cultivar			
118	HOR 13965	RBC118	*Hordeum sp.*	Australia	Australia	Cultivar			
119	HOR 4724	RBC119	*Hordeum vulgare* L. *convar. distichon var. nutans* (Rode) *Alef.*	East Europe	Armenia	Landrace			
120	BCC 1551	RBC120	*Hordeum vulgare* L. *convar. vulgare var. hybernum Viborg*	East Europe	Armenia	Cultivar			
121	HOR 4468	RBC121	*Hordeum vulgare* L. *convar. distichon var. nutans* (Rode) *Alef.*	East Europe	Armenia	Landrace			
122	HOR 7394	RBC122	*Hordeum vulgare* L. *convar. distichon var. syriacum* (Vavilov & Orlov) Mansf.	East Europe	Armenia	Landrace			
123	BCC 1474	RBC123	*Hordeum vulgare* L. *convar. vulgare var. hybernum Viborg*	East Europe	Ukraine	Cultivar			
124	BCC 1493	RBC124	*Hordeum vulgare* L. *convar. vulgare var. hybernum Viborg*	East Europe	Ukraine	Cultivar			
125	BCC 1505	RBC125	*Hordeum vulgare* L. *convar. vulgare var. hybernum Viborg*	East Europe	Ukraine	Cultivar			
126	BCC 1533	RBC126	*Hordeum vulgare* L. *convar. vulgare var. hybernum Viborg*	East Europe	Ukraine	Cultivar			
127	HOR 11017	RBC127	*Hordeum spontaneum* K.Koch	East Europe	Greece	Wild type	NN 20	35°30'59'' N	024°01'59'' E
128	HOR 10924	RBC128	*Hordeum spontaneum* K.Koch *var. spontaneum*	East Europe	Greece	Wild type	NN 32	28°58'54'' N	26°23'53'' E
129	HOR 1131	RBC129	*Hordeum vulgare* L. *convar. vulgare var. hybernum Viborg*	East Europe	Greece	Landrace	NN 226	35°27'52'' N	023°46'17'' E
130	HOR 12418	RBC130	*Hordeum spontaneum* K.Koch	East Europe	Greece	Wild type	NN 20	35°30'59'' N	024°01'59'' E
131	HOR 199	RBC131	*Hordeum vulgare* L. *convar. vulgare var. hybernum Viborg*	East Europe	Russia	Landrace			
132	HOR 3372	RBC132	*Hordeum vulgare* L. *convar. vulgare var. hybernum Viborg*	East Europe	Russia	Landrace			
133	HOR 2448	RBC133	*Hordeum vulgare* L. *convar. vulgare var. hybernum Viborg*	East Europe	Russia	Landrace			
134	BCC 1491	RBC134	*Hordeum vulgare* L. *convar. vulgare var. hybernum Viborg*	East Europe	Russia	Landrace			
135	ICB 181500	RBC135	*Hordeum vulgare ssp. vulgare*	Asia	Tadjikistan	Wild type	NN 1030	39°28'25''N	067°30'1''E
136	ICB 180063	RBC136	*Hordeum vulgare ssp. vulgare*	Middle East	Turkey	Wild type			
137	ICB 180070	RBC137	*Hordeum vulgare ssp. vulgare*	Middle East	Turkey	Wild type	NN 840	39°39'52''N	031°9'40''E

138	ICB 181162	RBC138	*Hordeum vulgare ssp. vulgare*	Middle East	Iran	Wild type			
139	CCS 004	RBC139	*Hordeum vulgare* L.	West Europe	Germany	Cultivar			
140	CCS 010	RBC140	*Hordeum vulgare* L.	West Europe	Germany	Cultivar			
141	CCS 012	RBC141	*Hordeum vulgare* L.	West Europe	Germany	Cultivar			
142	CCS 018	RBC142	*Hordeum vulgare* L.	West Europe	Germany	Cultivar			
143	CCS 023	RBC143	*Hordeum vulgare* L.	West Europe	Germany	Cultivar			
144	CCS 041	RBC144	*Hordeum vulgare* L.	West Europe	Germany	Cultivar			
145	CCS 052	RBC145	*Hordeum vulgare* L.	West Europe	Germany	Cultivar			
146	CCS 060	RBC146	*Hordeum vulgare* L.	West Europe	Germany	Cultivar			
147	CCS 067	RBC147	*Hordeum vulgare* L.	West Europe	Germany	Cultivar			
148	CCS 081	RBC148	*Hordeum vulgare* L.	West Europe	Germany	Cultivar			
149	CCS 083	RBC149	*Hordeum vulgare* L.	West Europe	Germany	Cultivar			
150	CCS 084	RBC150	*Hordeum vulgare* L.	West Europe	Germany	Cultivar			
151	CCS 086	RBC151	*Hordeum vulgare* L.	West Europe	Germany	Cultivar			
152	CCS 089	RBC152	*Hordeum vulgare* L.	West Europe	Germany	Cultivar			
153	CCS 095	RBC153	*Hordeum vulgare* L.	West Europe	Germany	Cultivar			
154	CCS 096	RBC154	*Hordeum vulgare* L.	West Europe	Germany	Cultivar			
155	CCS 109	RBC155	*Hordeum vulgare* L.	West Europe	Germany	Cultivar			
156	CCS 121	RBC156	*Hordeum vulgare* L.	West Europe	Germany	Cultivar			
157	CCS 141	RBC157	*Hordeum vulgare* L.	West Europe	Germany	Cultivar			
158	Agueda	RBC158	*Hordeum vulgare* L.	West Europe	Germany	Cultivar			
159	Montoya	RBC159	*Hordeum vulgare* L.	West Europe	Germany	Cultivar			
160	Danielle	RBC160	*Hordeum vulgare* L.	West Europe	Germany	Cultivar			
161	Britney	RBC161	*Hordeum vulgare* L.	West Europe	Germany	Cultivar			
162	Andreia	RBC162	*Hordeum vulgare* L.	West Europe	Germany	Cultivar			
163	Mutante	RBC163	*Hordeum vulgare* L.	West Europe	Germany	Cultivar			
164	BCC 1348	RBC164	*Hordeum vulgare* L. *convar. vulgare var. hybernum Viborg*	West Europe	Spain	Cultivar			
165	BCC 1523	RBC165	*Hordeum vulgare* L. *convar. vulgare var. hybernum Viborg*	West Europe	Spain	Cultivar			
166	HOR 19267	RBC166	*Hordeum sp.*	West Europe	Spain	Landrace			
167	BCC 1586	RBC167	*Hordeum vulgare* L.	West Europe	Spain	Cultivar			
168	HOR 873	RBC168	*Hordeum vulgare* L. *convar. vulgare var. hybernum Viborg*	West Europe	France	Cultivar			
169	HOR 1132	RBC169	*Hordeum vulgare* L. *convar. vulgare var. hybernum Viborg*	West Europe	France	Landrace	NN 650m	42°27'55" N	2°54'49" E
170	BCC 1380	RBC170	*Hordeum vulgare* L. *convar. distichon var. nutans* (Rode) *Alef.*	West Europe	France	Cultivar			
171	HOR 11790	RBC171	*Hordeum vulgare* L. *convar. distichon var. nutans* (Rode) *Alef.*	West Europe	France	Cultivar			
172	BCC 829	RBC172	*Hordeum vulgare* L.	West Europe	GB/Irland	Cultivar			
173	HOR 16665	RBC173	*Hordeum sp.*	West Europe	GB/Irland	Cultivar			
174	HOR 18101	RBC174	*Hordeum sp.*	West Europe	GB/Irland	Landrace			
175	HOR 12047	RBC175	*Hordeum vulgare* L. *convar. vulgare var. hybernum Viborg*	West Europe	GB/Irland	Landrace	NN 157	52°24'28" N	001°56'41" W

176	NGB8822	RBC176	*Hordeum vulgare ssp. vulgare*	West Europe	GB/Irland	Landrace			
177	NGB9480	RBC177	*Hordeum vulgare ssp. vulgare*	West Europe	GB/Irland	Landrace			
178	NGB4605	RBC178	*Hordeum vulgare ssp. vulgare*	West Europe	Romania	Landrace			
179	NGB9312	RBC179	*Hordeum vulgare ssp. vulgare*	West Europe	Romania	Landrace			
