# Supplementary material for: Genome-Wide Association Mapping in the Global Diversity Set Reveals New QTL Controlling Root System and Related Shoot Variation in Barley
Source: Front Plant Sci. 2016 Jul 19;7:1061. doi: 10.3389/fpls.2016.01061 (PMC4949209; doi:10.3389/fpls.2016.01061)
Supplement: Supplementary file 2 [file Table_2.PDF]

Table S2. Variance analysis for five analyzed traits among 179 accessions in 2014 and 2015 under control and drought conditions

Trait	SOV	DF	MS	F value	P value	H^2
**Rdw**	Treatment	1	8918.52	1154.98	<0.001	0.62
	$\text{Replication}_{(\text{Treatment})}$	6	12.47	1.61	ns	
	Genotype	177	35.09	4.54	<0.001	
	Genotype x Treatment	177	11.44	1.48	<0.001	
	Genotype x Year	173	39.70	5.14	<0.001	
	Genotype x Treatment x Year	171	10.10	1.31	<0.01	
**Rl**	Treatment	1	15701.64	397.44	<0.001	0.48
	$\text{Replication}_{(\text{Treatment})}$	6	343.37	8.69	<0.001	
	Genotype	177	94.85	2.40	<0.001	
	Genotype x Treatment	177	38.14	0.97	ns	
	Genotype x Year	173	134.55	3.41	<0.001	
	Genotype x Treatment x Year	171	60.94	1.54	<0.001	
**Sdw**	Treatment	1	112422.61	5212.17	<0.001	0.54
	$\text{Replication}_{(\text{Treatment})}$	6	376.65	17.46	<0.001	
	Genotype	177	115.48	5.35	<0.001	
	Genotype x Treatment	177	38.14	1.77	<0.001	
	Genotype x Year	173	206.48	9.57	<0.001	
	Genotype x Treatment x Year	171	29.19	1.35	<0.01	
**Til**	Treatment	1	17938.82	1901.97	<0.001	0.90
	$\text{Replication}_{(\text{Treatment})}$	6	58.34	6.19	<0.001	
	Genotype	177	395.32	41.91	<0.001	
	Genotype x Treatment	177	25.75	2.73	<0.001	
	Genotype x Year	173	275.03	29.16	<0.001	
	Genotype x Treatment x Year	171	11.50	1.22	<0.05	
**RS**	Treatment	1	81.14	398.38	<0.001	0.66
	$\text{Replication}_{(\text{Treatment})}$	6	0.26	1.30	ns	
	Genotype	177	1.18	5.78	<0.001	
	Genotype x Treatment	177	0.31	1.53	<0.001	
	Genotype x Year	173	0.44	2.17	<0.001	
	Genotype x Treatment x Year	171	0.30	1.48	<0.001	

Trait RDW = Root dry weight, RL = Root length, SDW = Shoot dry weight, Til = No of tiller, RS = Root-shoot ratio, SOV = Sources of variation, DF = Degrees of freedom, MS = Mean sum of squares, P-value = indicates the level of significance at 0.05, 0.01 and 0.001, ns: non-significant, H^2 = Heritability
