# Supplementary material for: Genome-Wide Association Mapping in the Global Diversity Set Reveals New QTL Controlling Root System and Related Shoot Variation in Barley
Source: Front Plant Sci. 2016 Jul 19;7:1061. doi: 10.3389/fpls.2016.01061 (PMC4949209; doi:10.3389/fpls.2016.01061)
Supplement: Supplementary file 3 [file Table_3.PDF]

Table S3. Pearson correlation coefficients between root and shoot traits under control and drought conditions

	Trait	Rdw	Rl	RS	Sdw	Til
**Control**	**Rdw**	1				
	**Rl**	0.11**	1			
	**RS**	0.80***	0.18***	1		
	**Sdw**	ns	-0.13***	-0.53***	1	
	**Til**	0.39***	0.14***	0.54***	0.45***	1
**Stress**	**Rdw**	1				
	**Rl**	0.14***	1			
	**RS**	0.42***	0.13***	1		
	**Sdw**	0.16***	ns	-0.47***	1	
	**Til**	0.49***	0.25***	0.46***	0.23***	1

Trait: RDW = Root dry weight, RL = Root length, RS = Root-shoot ratio, SDW = Shoot dry weight, Til = No of tiller; *, **, *** = indicates the level of significance at 0.05 (*), 0.01 (**) and 0.001 (***), ns: non-significant
