# Supplementary material for: Genome-Wide Association Mapping in the Global Diversity Set Reveals New QTL Controlling Root System and Related Shoot Variation in Barley
Source: Front Plant Sci. 2016 Jul 19;7:1061. doi: 10.3389/fpls.2016.01061 (PMC4949209; doi:10.3389/fpls.2016.01061)
Supplement: Supplementary file 4 [file Table_4.PDF]

Table S4: Order of genotypes and allele distribution across individual QTL pin plots.

Accession	Origin	QRdw.5H		QSdw.2H.b		QTil.1H		QRS.5H	
		Position x-axis*	Allele	Position x-axis*	Allele	Position x-axis*	Allele	Position x-axis*	Allele
ICB 180215	Turkmenistan	175	A/A	18	T/T	147	A/A	172	G/G
IG 124000	Uzbekistan	174	A/A	31	T/T	169	A/A	166	A/A
HOR 11017	Greece	173	A/A	16	T/T	172	A/A	170	A/A
BCC 1551	Armenia	172	G/G	23	T/T	160	G/G	168	G/G
HOR 2514	India	171	A/A	146	T/T	151	A/A	140	A/A
ICB 180117	Palestine	170	A/A	3	T/T	175	A/A	175	A/A
HOR 19883	Turkey	169	A/A	4	T/T	174	G/G	174	A/A
HOR 12047	GB/Irland	168	A/A	29	T/T	137	G/G	164	A/A
BCC 776	Nepal	167	A/A	53	T/C	132	G/G	159	A/A
ICB 180006	Syria	166	G/G	10	C/C	167	A/A	165	G/G
HOR 19267	Spain	165	G/A	6	T/T	158	G/G	169	G/A
ICB 180410	Palestine	164	G/G	59	T/T	138	A/A	148	G/G
HOR 11790	France	163	A/A	8	T/T	159	G/G	173	A/A
ICB 181498	Uzbekistan	162	A/A	15	T/T	163	A/A	163	A/A
ICB 180092	Palestine	161	A/A	54	T/T	123	A/A	153	A/A
HOR 2448	Russia	160	A/A	7	T/T	170	G/G	171	A/A
HOR 9840	Libya	159	A/A	35	T/C	161	A/A	156	A/A
HOR 9721	Libya	158	G/G	38	T/T	173	A/A	149	G/G
HOR 12418	Greece	157	A/A	2	T/T	157	A/A	167	A/A
HOR 8372	India	156	G/G	49	C/C	153	G/G	150	G/G
HOR 4124	Mexico	155	G/A	60	T/T	150	G/G	152	G/A
HOR 1566	China	154	G/G	118	T/T	71	G/G	127	G/G
HOR 16665	GB/Irland	153	G/G	100	T/T	117	G/G	142	G/G
IG 124017	Uzbekistan	152	A/A	21	T/T	146	A/A	162	A/A
BCC 881	Canada	151	G/G	173	C/C	44	G/G	87	G/G
HOR 7449	Peru	150	G/G	101	T/T	16	G/G	136	G/G
HOR 17616	Nepal	149	A/A	36	T/T	140	G/G	157	A/A
ICB 180862	Syria	148	G/G	20	T/T	148	A/A	160	G/G
CCS 083	Germany	147	G/G	164	T/T	126	G/G	118	G/G
Mutante	Germany	146	G/G	88	T/T	115	G/G	123	G/G
BCC 1493	Ukraine	145	G/G	130	T/T	56	G/G	108	G/G
HOR 16359	Sudan	144	G/G	33	T/T	127	G/G	155	G/G
ICB 180508	Israel	143	G/G	17	T/T	164	A/A	154	G/G
BCC 282	Uzbekistan	142	G/G	11	T/T	155	G/G	158	G/G
ICB 181160	Iran	141	G/G	56	T/T	92	A/A	133	G/G
HOR 8367	India	140	G/G	84	T/T	36	G/G	119	G/G
NGB6952	Afghanistan	139	G/G	27	T/C	108	G/G	151	G/G
BCC 871	Chile	138	G/G	131	T/T	94	G/G	124	G/G
NGB9312	Romania	137	G/G	147	T/T	42	G/G	91	G/G
ICB 180070	Turkey	136	G/G	104	T/T	152	A/A	141	G/G
HOR 10924	Greece	135	G/G	14	T/T	156	A/A	161	G/G
HOR 13965	Australia	134	G/G	86	T/C	31	G/G	125	G/G

CCS 089	Germany	133	G/G	145	T/T	85	G/G	98	G/G
HOR 20110	Chile	132	G/G	78	T/T	40	G/G	122	G/G
ICB 180013	Jordan	131	A/A	45	T/T	141	A/A	137	A/A
CCS 084	Germany	130	G/G	70	T/T	121	G/G	134	G/G
ICB 180217	Turkmenistan	129	A/A	37	T/T	135	A/A	139	A/A
HOR 2981	Chile	128	G/G	73	T/T	7	G/G	132	G/G
BCC 844	Colombia	127	G/A	159	T/T	39	G/G	68	G/A
HOR 17307	Uruguay	126	G/G	123	T/T	61	G/G	102	G/G
HOR 14485	Chile	125	G/G	106	T/C	20	G/G	114	G/G
BCC 1523	Spain	124	A/A	67	C/C	34	G/G	109	A/A
ICB 180994	Palestine	123	G/G	42	T/T	149	A/A	129	G/G
CCS 086	Germany	122	G/G	121	T/T	131	G/G	96	G/G
HOR 9565	Peru	121	G/G	157	T/T	41	G/G	66	G/G
ICB 180260	Israel	120	G/G	48	T/T	154	G/G	144	G/G
BCC 891	USA	119	G/G	95	T/T	111	G/G	107	G/G
NGB8872	Afghanistan	118	G/G	166	C/C	78	G/G	73	G/G
ICB 181492	Turkmenistan	117	G/G	44	T/T	162	A/A	138	G/G
BCC 801	Canada	116	G/G	114	T/T	118	G/G	106	G/G
CCS 067	Germany	115	G/G	93	T/T	119	G/G	111	G/G
HOR 13597	Mexico	114	G/G	153	C/C	11	G/G	76	G/G
CCS 052	Germany	113	G/G	103	T/T	101	G/G	120	G/G
HOR 4206	Australia	112	A/A	152	C/C	52	G/G	77	A/A
BCC 882	Bolivia	111	G/G	138	T/T	29	G/G	85	G/G
BCC 1380	France	110	G/G	87	T/T	112	G/G	100	G/G
NGB9599	Afghanistan	109	G/G	50	T/C	50	G/G	117	G/G
ICB 181500	Tadjikistan	108	A/A	40	T/T	144	A/A	130	A/A
BCC 921	Colombia	107	G/A	32	T/T	139	G/G	145	G/A
CCS 081	Germany	106	G/G	155	T/T	84	G/G	61	G/G
CCS 004	Germany	105	G/G	41	T/T	130	G/G	131	G/G
CCS 121	Germany	104	G/G	105	T/T	76	G/G	101	G/G
CCS 012	Germany	103	G/G	39	T/T	122	G/G	128	G/G
HOR 13412	Marocco	102	G/G	127	T/C	19	G/G	75	G/G
CCS 023	Germany	101	G/G	58	T/T	105	G/G	143	G/G
ICB 180069	Iraq	100	A/A	77	T/T	145	A/A	116	A/A
BCC 862	Uruguay	99	G/G	172	C/C	25	G/G	39	G/G
HOR 18101	GB/Irland	98	G/G	91	C/C	113	G/G	121	G/G
BCC 1474	Ukraine	97	G/G	99	T/T	63	G/G	97	G/G
CCS 096	Germany	96	G/G	142	T/T	102	G/G	72	G/G
HOR 56	China	95	G/A	79	T/C	43	G/G	113	G/A
Agueda	Germany	94	G/G	22	T/T	109	G/G	135	G/G
BCC 126	Marocco	93	G/G	126	T/C	60	G/G	71	G/G
BCC 875	USA	92	G/G	156	C/C	28	G/G	58	G/G
HOR 873	France	91	G/G	132	C/C	49	G/G	81	G/G
ICB 180211	Turkmenistan	90	A/A	13	T/T	171	A/A	146	A/A
BCC 906	USA	89	G/G	89	C/C	10	G/G	92	G/G
HOR 7443	Bolivia	88	G/G	160	T/T	67	G/G	41	G/G

CCS 095	Germany	87	G/G	133	T/T	83	G/G	94	G/G
BCC 149	Marocco	86	G/G	119	T/C	17	G/G	83	G/G
ICB 181268	Jordan	85	A/A	30	C/C	142	G/G	110	A/A
NGB9480	GB/Irland	84	G/G	175	C/C	99	G/G	14	G/G
HOR 1132	France	83	G/G	66	T/C	2	G/G	93	G/G
BCC 852	Canada	82	G/G	136	C/C	57	G/G	86	G/G
BCC 927	Peru	81	G/G	144	T/T	26	G/G	57	G/G
BCC 613	Japan	80	G/A	165	C/C	88	G/G	36	G/A
NGB4673	Afghanistan	79	G/G	171	T/C	73	G/G	15	G/G
BCC 1586	Spain	78	G/G	116	C/C	3	G/G	65	G/G
HOR 9838	Libya	77	G/G	46	C/C	37	G/G	112	G/G
ICB 181243	Pakistan	76	G/G	150	T/T	124	A/A	35	G/G
ICB 180007	Jordan	75	A/A	43	T/T	165	A/A	115	A/A
NGB9606	Afghanistan	74	G/G	109	T/C	38	G/G	82	G/G
HOR 4724	Armenia	73	G/G	161	C/C	104	G/G	49	G/G
Andreia	Germany	72	G/G	163	C/C	110	G/G	32	G/G
CCS 010	Germany	71	G/G	69	T/T	125	G/G	103	G/G
HOR 2589	Sudan	70	G/G	162	C/C	12	G/G	28	G/G
BCC 1348	Spain	69	G/A	76	T/C	116	G/G	88	G/A
HOR 20921	Israel	68	G/A	112	T/C	24	G/G	60	G/A
NGB8822	GB/Irland	67	G/G	125	C/C	91	G/G	54	G/G
HOR 7446	Bolivia	66	G/G	149	T/C	45	G/G	34	G/G
BCC 928	Bolivia	65	G/G	97	T/T	95	G/G	105	G/G
BCC 896	Uruguay	64	G/G	151	C/C	13	G/G	67	G/G
Britney	Germany	63	G/G	143	C/C	133	G/G	52	G/G
HOR 35	Australia	62	G/G	140	C/C	21	G/G	45	G/G
ICB 181162	Iran	61	A/A	83	T/T	89	A/A	84	A/A
HOR 14936	Turkey	60	G/G	111	T/C	107	G/G	62	G/G
BCC 888	Canada	59	G/G	141	C/C	35	G/G	56	G/G
HOR 2684	Iran	58	G/G	168	T/T	136	G/A	12	G/G
ICB 180046	Iraq	57	A/A	9	T/T	168	A/A	147	A/A
CCS 060	Germany	56	G/G	68	C/C	98	G/G	89	G/G
Danielle	Germany	55	G/G	62	C/C	82	G/G	90	G/G
HOR 3372	Russia	54	G/G	158	C/C	100	G/G	21	G/G
HOR 11106	Iraq	53	G/G	85	T/T	14	G/G	70	G/G
HOR 16287	Sudan	52	G/G	71	T/T	128	G/G	95	G/G
BCC 829	GB/Irland	51	G/G	174	C/C	64	G/G	9	G/G
HOR 2692	Iran	50	G/G	169	T/T	120	G/A	27	G/G
HOR 199	Russia	49	G/G	122	C/C	59	G/G	55	G/G
HOR 10845	Colombia	48	G/G	98	C/C	51	G/G	79	G/G
HOR 19577	Peru	47	G/G	94	C/C	9	G/G	78	G/G
HOR 930	Turkey	46	G/G	167	C/C	65	G/G	8	G/G
BCC 848	Mexico	45	G/G	135	T/T	18	G/G	26	G/G
HOR 15956	Sudan	44	G/G	124	T/T	77	G/G	44	G/G
BCC 732	Nepal	43	G/G	92	C/C	68	G/G	64	G/G
HOR 10843	Colombia	42	G/G	108	C/C	1	G/G	40	G/G

HOR 1479	China	41	G/G	64	T/T	79	G/G	104	G/G
HOR 7599	Pakistan	40	G/G	154	C/C	53	G/G	10	G/G
HOR 11421	India	39	G/G	134	C/C	66	G/G	59	G/G
HOR 7603	Pakistan	38	G/G	129	C/C	69	G/G	23	G/G
HOR 14953	Turkey	37	G/G	51	T/C	74	G/G	69	G/G
BCC 1491	Russia	36	G/G	170	C/C	62	G/G	6	G/G
BCC 131	Marocco	35	G/G	139	T/C	46	G/G	22	G/G
ICB 180902	Syria	34	G/G	24	T/T	106	A/A	99	G/G
HOR 20173	Australia	33	G/G	120	T/T	81	G/G	16	G/G
HOR 16097	Egypt	32	G/G	148	C/C	23	G/G	30	G/G
BCC 1533	Ukraine	31	G/G	137	T/T	27	G/G	17	G/G
ICB 181442	Jordan	30	G/G	82	T/T	134	A/A	42	G/G
HOR 1131	Greece	29	G/G	102	T/C	30	G/G	37	G/G
HOR 16345	Uruguay	28	G/G	107	T/T	47	G/G	29	G/G
HOR 1510	China	27	G/G	19	T/T	5	G/G	126	G/G
HOR 10164	Libya	26	G/A	81	T/T	75	G/A	38	G/A
CCS 109	Germany	25	G/G	128	T/T	129	G/G	25	G/G
HOR 18209	Australia	24	G/G	117	C/C	58	G/G	20	G/G
IG 121857	Syria	23	G/G	26	T/T	114	A/A	80	G/G
BCC 1505	Ukraine	22	G/G	80	C/C	70	G/G	63	G/G
HOR 19027	Egypt	21	G/G	61	T/T	93	G/G	46	G/G
NGB4668	Afghanistan	20	G/G	55	C/C	55	G/G	53	G/G
ICB 181418	Jordan	19	G/G	90	T/T	166	A/A	19	G/G
CCS 018	Germany	18	G/G	34	T/T	96	G/G	74	G/G
BCC 817	USA	17	G/G	63	C/C	15	G/G	47	G/G
HOR 7394	Armenia	16	G/G	113	C/C	90	G/G	7	G/G
Montoya	Germany	15	G/G	72	C/C	86	G/G	18	G/G
HOR 19848	Japan	14	G/G	1	T/T	6	G/G	5	G/G
HOR 18945	Nepal	13	G/G	47	T/T	32	G/G	51	G/G
HOR 20117	Egypt	12	G/G	65	T/T	8	G/G	31	G/G
ICB 180329	Israel	11	G/G	28	T/T	143	A/A	48	G/G
NGB4605	Romania	10	G/G	115	C/C	33	G/G	4	G/G
HOR 16714	China	9	G/G	57	C/C	54	G/G	24	G/G
HOR 15779	Japan	8	G/G	74	T/T	80	G/G	11	G/G
HOR 19308	Egypt	7	G/G	52	T/T	48	G/G	13	G/G
ICB 180063	Turkey	6	G/G	110	T/T	22	A/A	2	G/G
HOR 4468	Armenia	5	G/G	96	C/C	103	G/G	1	G/G
HOR 18647	Japan	4	G/G	5	T/T	4	G/G	43	G/G
HOR 18401	Pakistan	3	G/G	12	C/C	72	G/G	50	G/G
HOR 2687	Iran	2	G/G	25	T/C	87	G/A	3	G/G
CCS 041	Germany	1	G/G	75	T/T	97	G/G	33	G/G

*Individual genotypes can be found on the x-axis with indicated numbers starting from 1 in each pin plot
