# Supplementary material for: Genome-Wide Association Mapping in the Global Diversity Set Reveals New QTL Controlling Root System and Related Shoot Variation in Barley
Source: Front Plant Sci. 2016 Jul 19;7:1061. doi: 10.3389/fpls.2016.01061 (PMC4949209; doi:10.3389/fpls.2016.01061)
Supplement: Supplementary file 5 [file Presentation_1.PDF]

Supplementary Material

High resolution association mapping reveals new QTL mediating root and shoot variation in a global barley diversity set

Stephan Reinert , Annika Kortz, Jens Léon, Ali Ahmad Naz *

*** Correspondence:** Corresponding Author: a.naz@uni-bonn.de

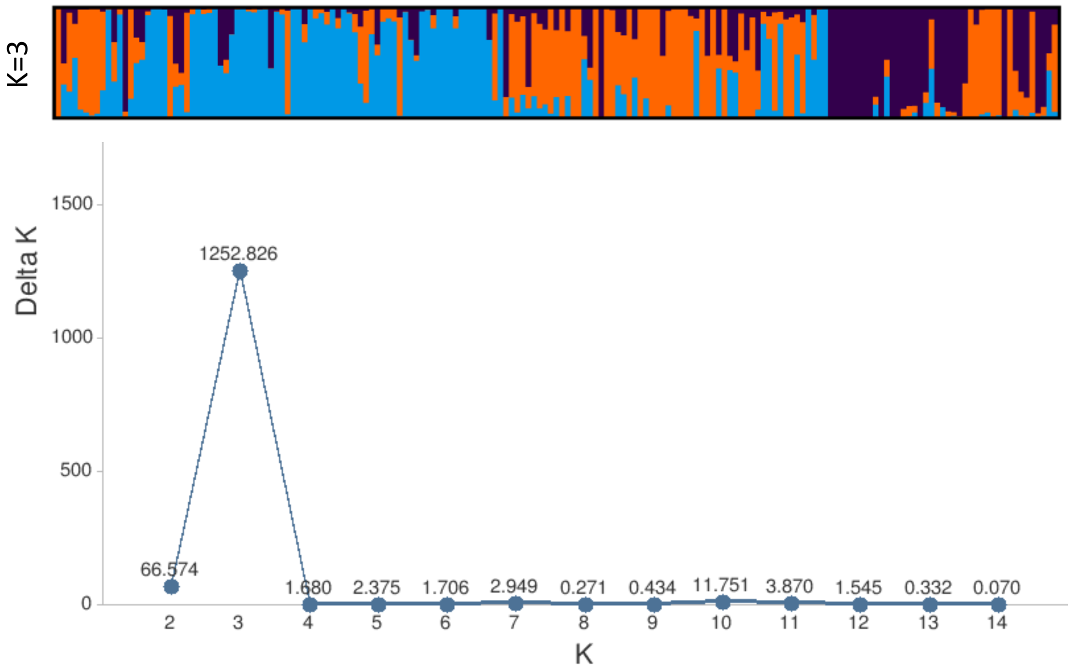

Figure S1. Population structure and genetic differentiation analysis for barley diversity panel. Population structure of 179 accessions calculated with 5892 polymorphic SNP marker revealed three sub-cluster (K=3). The genetic distribution within each accession is denoted as a colored vertical line. The three different colors represent different sub-clusters.

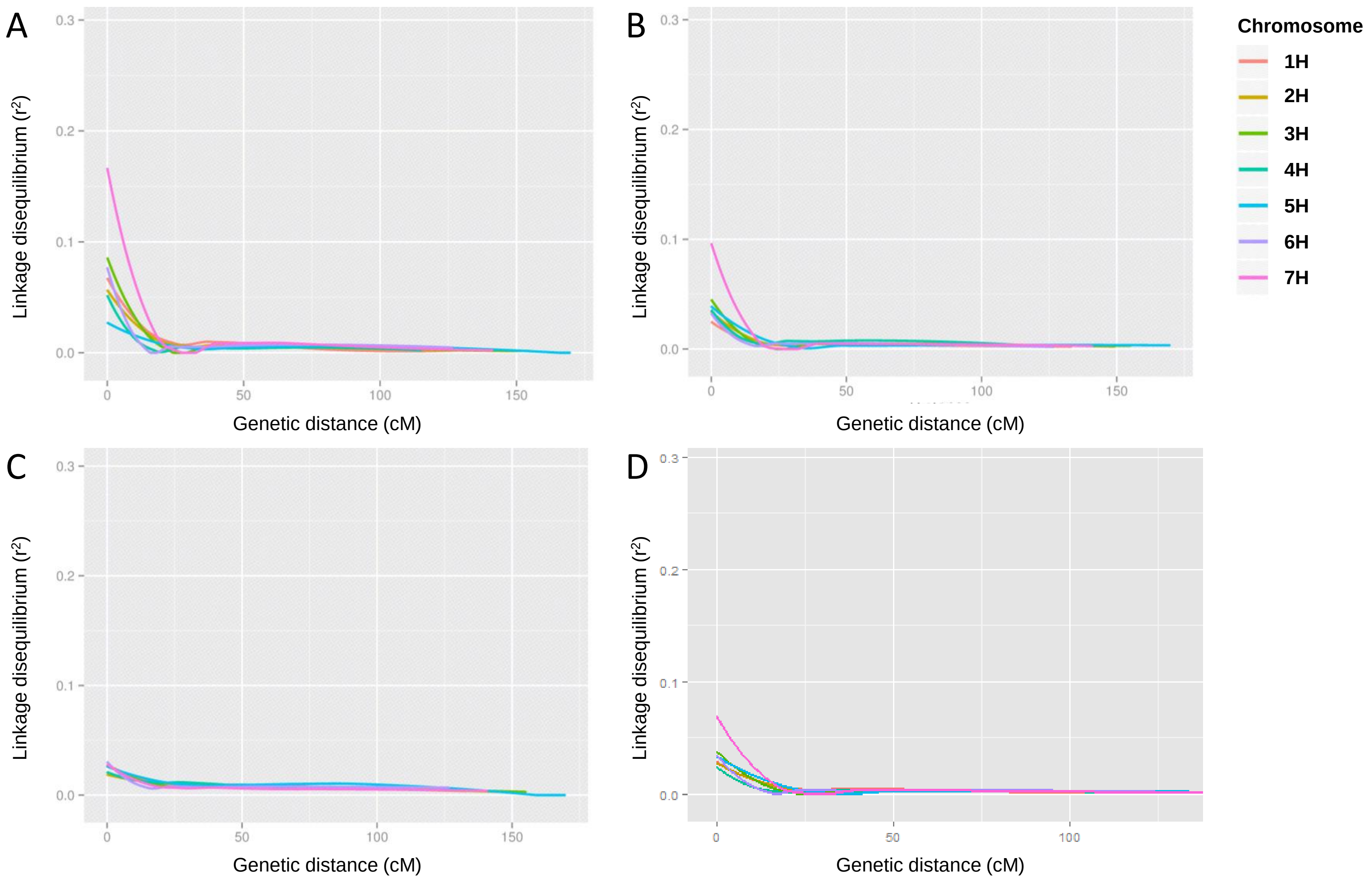

Figure S2: Plot of LD-decay for the global barley population with 5892 SNP marker. The colored lines represent the seven chromosomes (Chr) of barley. A) Plot of LD-decay for barley cultivars. B) Plot of LD-decay for landraces. C) Plot of LD-decay for wild barley lines. D) Plot of LD-decay for the whole global population.

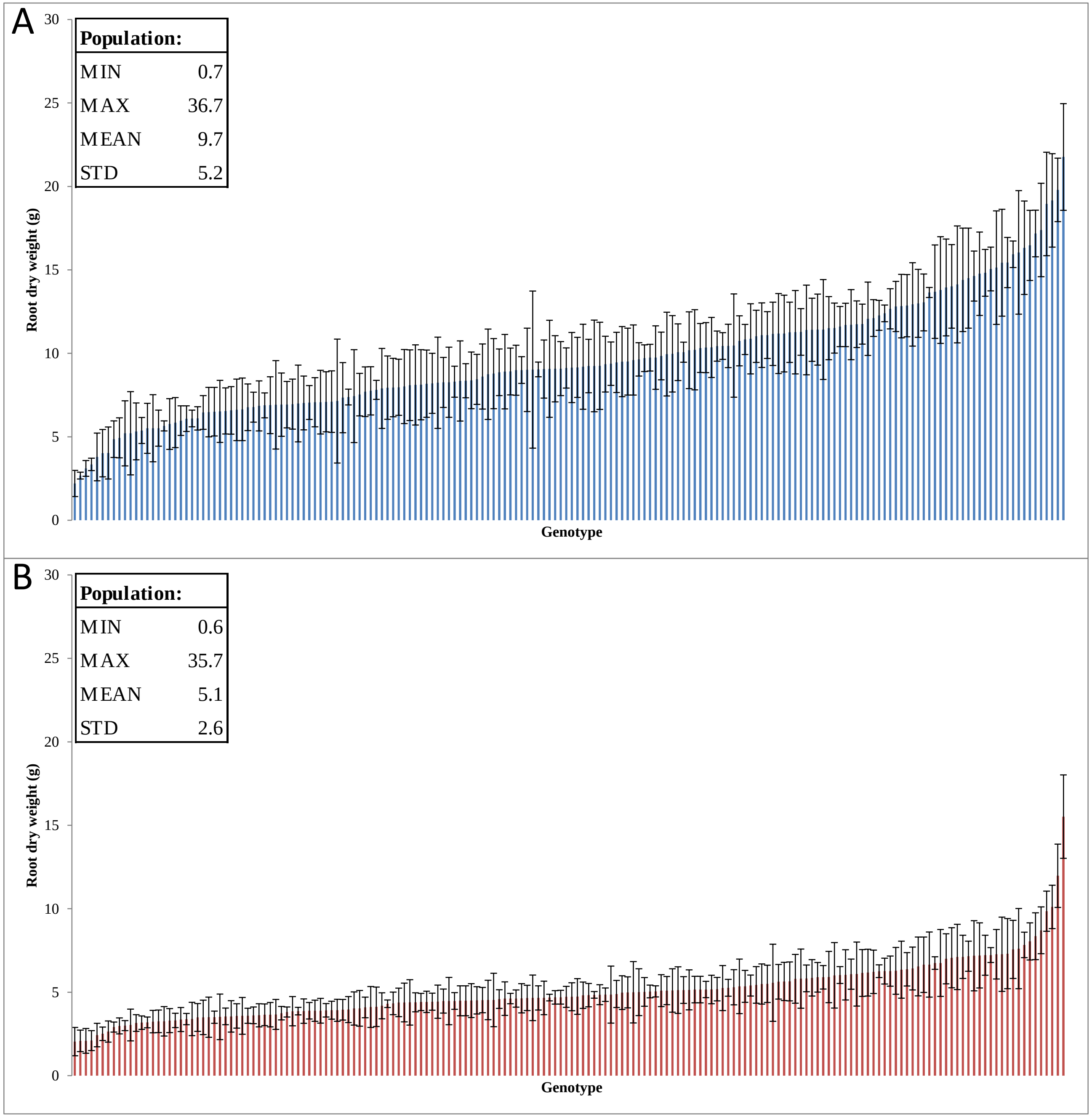

Figure S3: Phenotypic variation of root dry weight (Rdw) in 2014 of global population under control and drought conditions. A) Rdw (g) of genotypes of barley population under control conditions as well as population mean, max, min and standard deviation (STD). B) Rdw (g) of genotypes of barley population under drought conditions as well as population mean, max, min and standard deviation (STD). N = 4

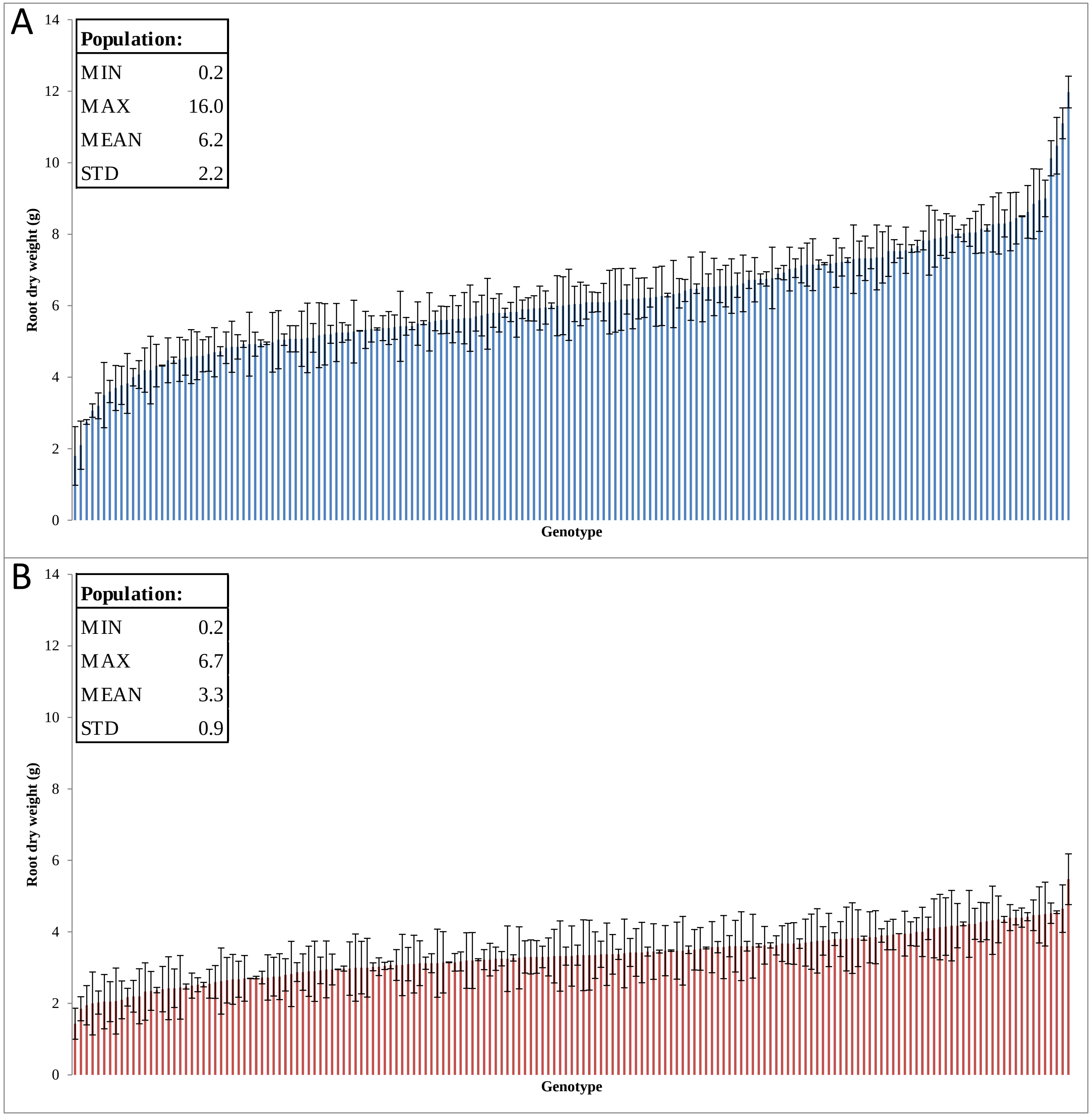

Figure S4: Phenotypic variation of root dry weight (Rdw) in 2015 of global population under control and drought conditions. A) Rdw (g) of genotypes of barley population under control conditions as well as population mean, max, min and standard deviation (STD). B) Rdw (g) of genotypes of barley population under drought conditions as well as population mean, max, min and standard deviation (STD). N = 4

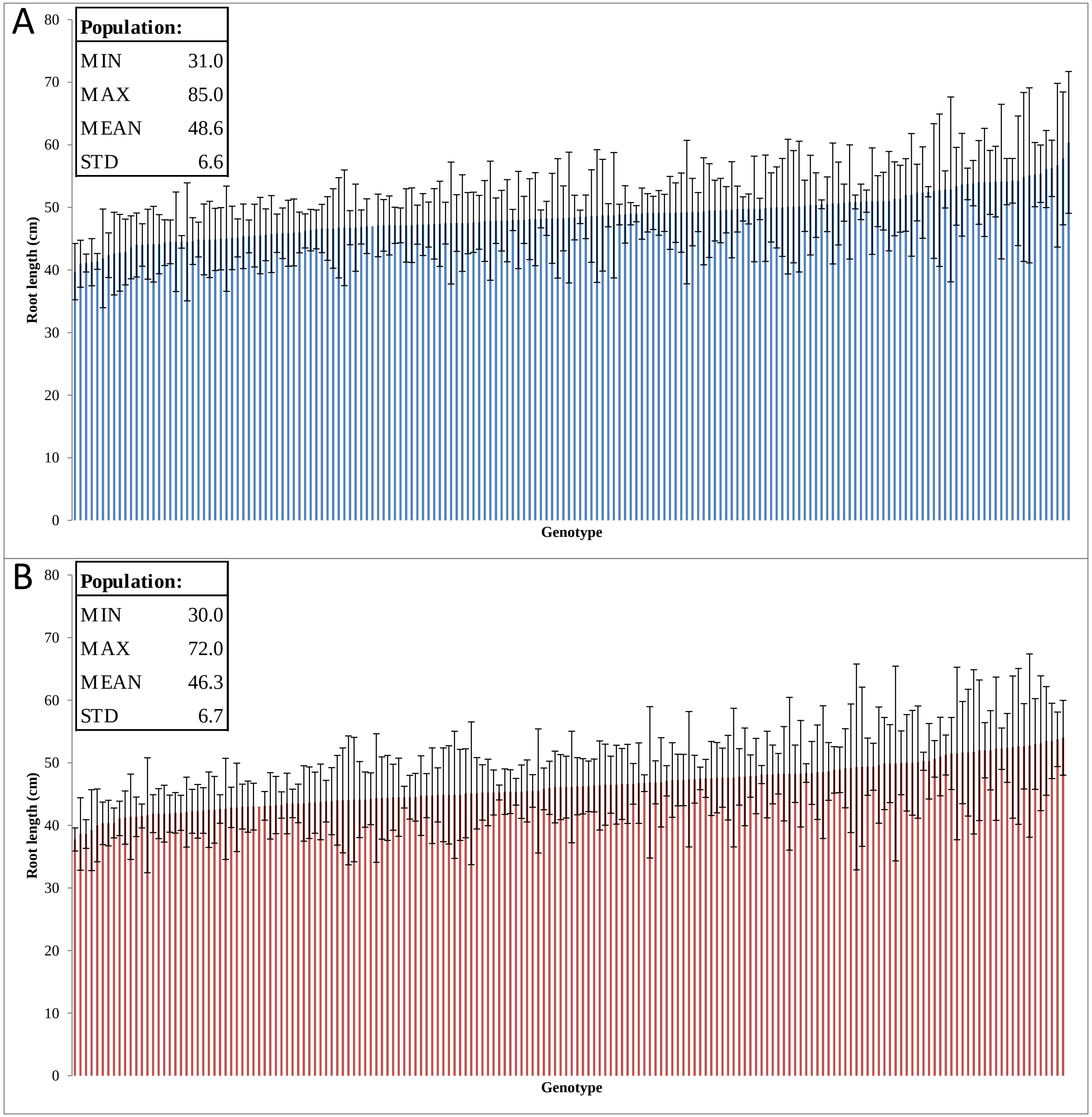

Figure S5: Phenotypic variation of root length (Rl) in 2014 of global population under control and drought conditions. A) Rl (cm) of genotypes of barley population under control conditions as well as population mean, max, min and standard deviation (STD). B) Rl (cm) of genotypes of barley population under drought conditions as well as population mean, max, min and standard deviation (STD). N = 4

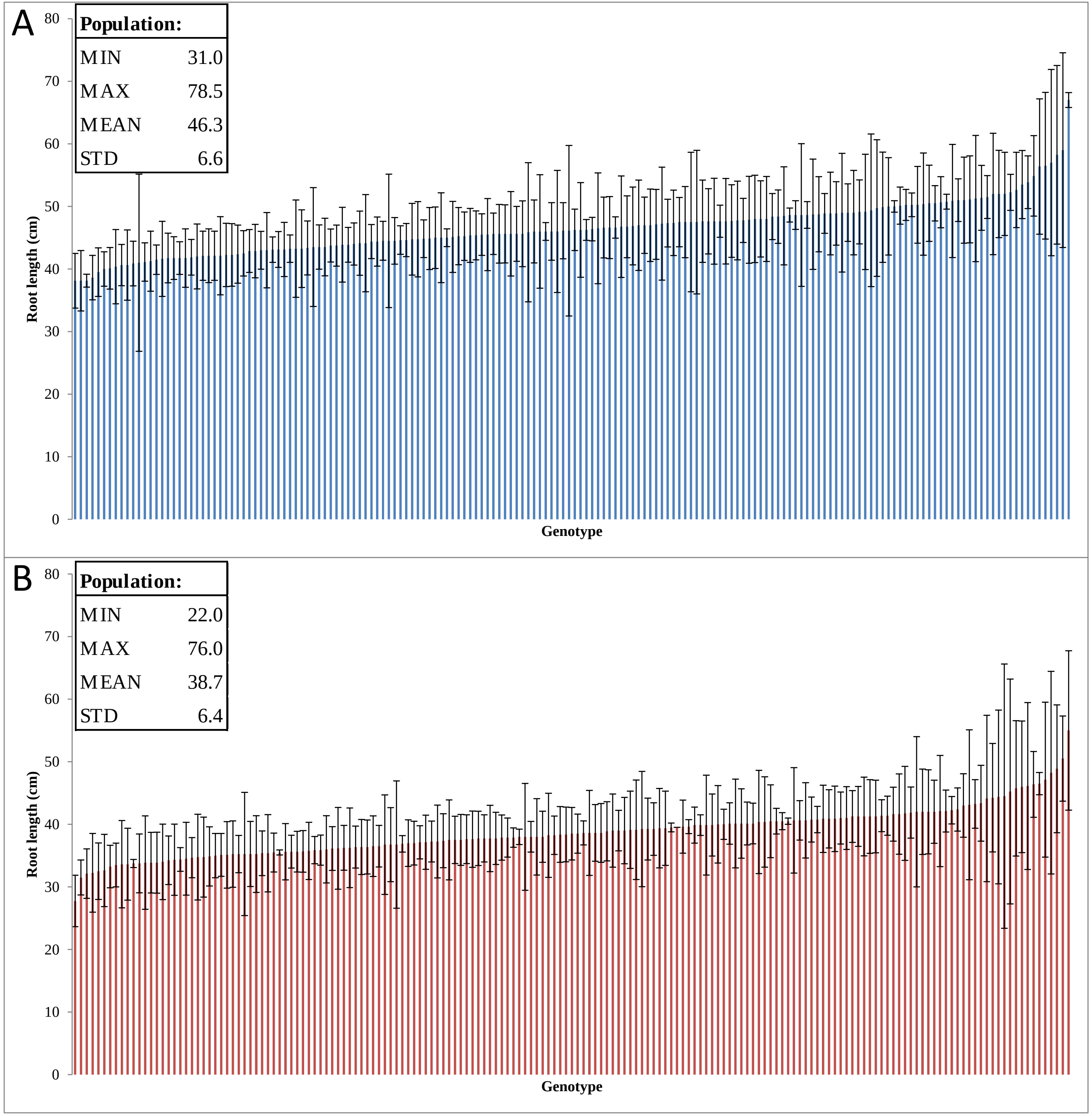

Figure S6: Phenotypic variation of root length (Rl) in 2015 of global population under control and drought conditions. A) Rl (cm) of genotypes of barley population under control conditions as well as population mean, max, min and standard deviation (STD). B) Rl (cm) of genotypes of barley population under drought conditions as well as population mean, max, min and standard deviation (STD). N = 4

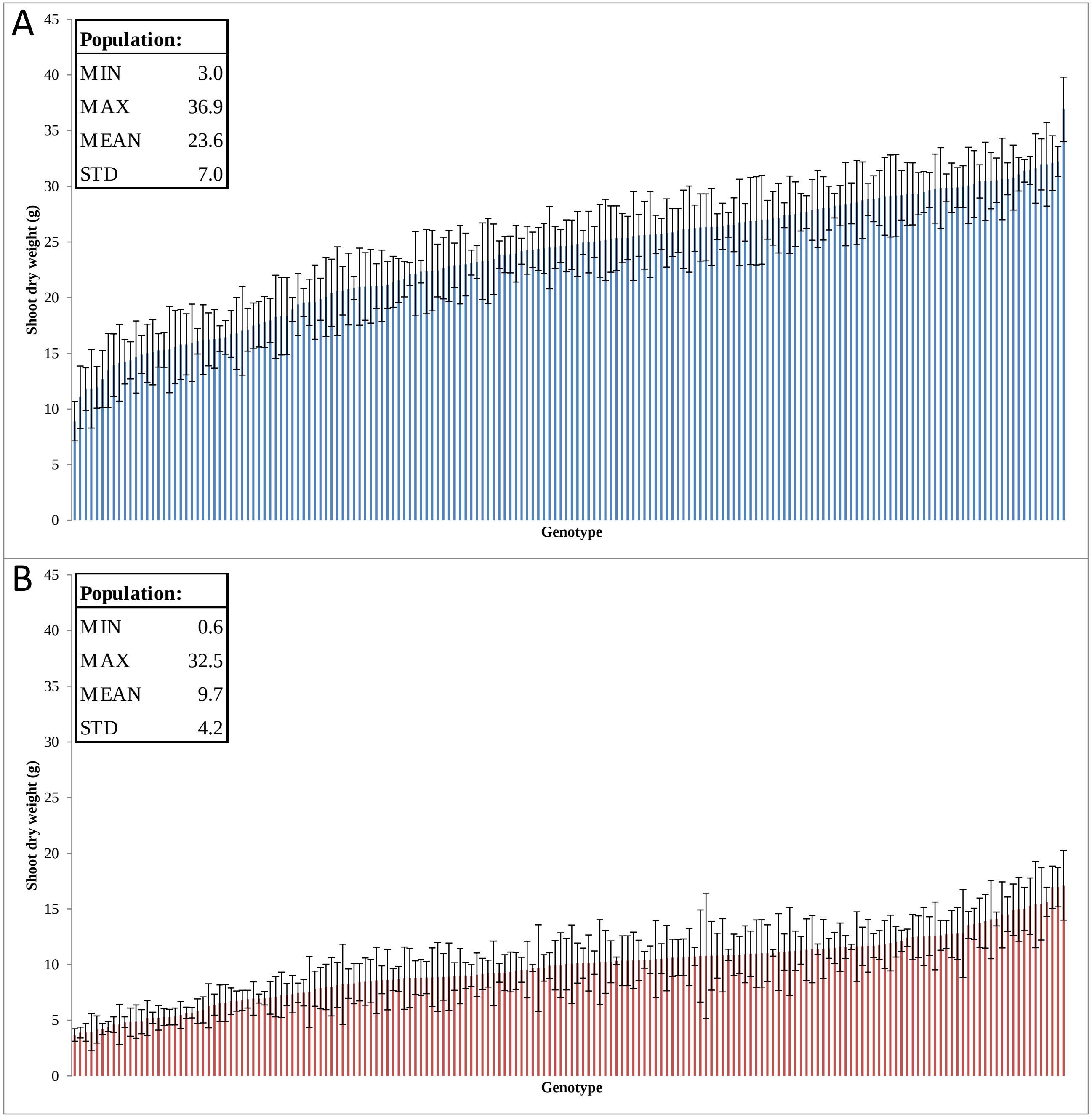

Figure S7: Phenotypic variation of shoot dry weight (Sdw) in 2014 of global population under control and drought conditions. A) Sdw (g) of genotypes of barley population under control conditions as well as population mean, max, min and standard deviation (STD). B) Sdw (g) of genotypes of barley population under drought conditions as well as population mean, max, min and standard deviation (STD). N = 4

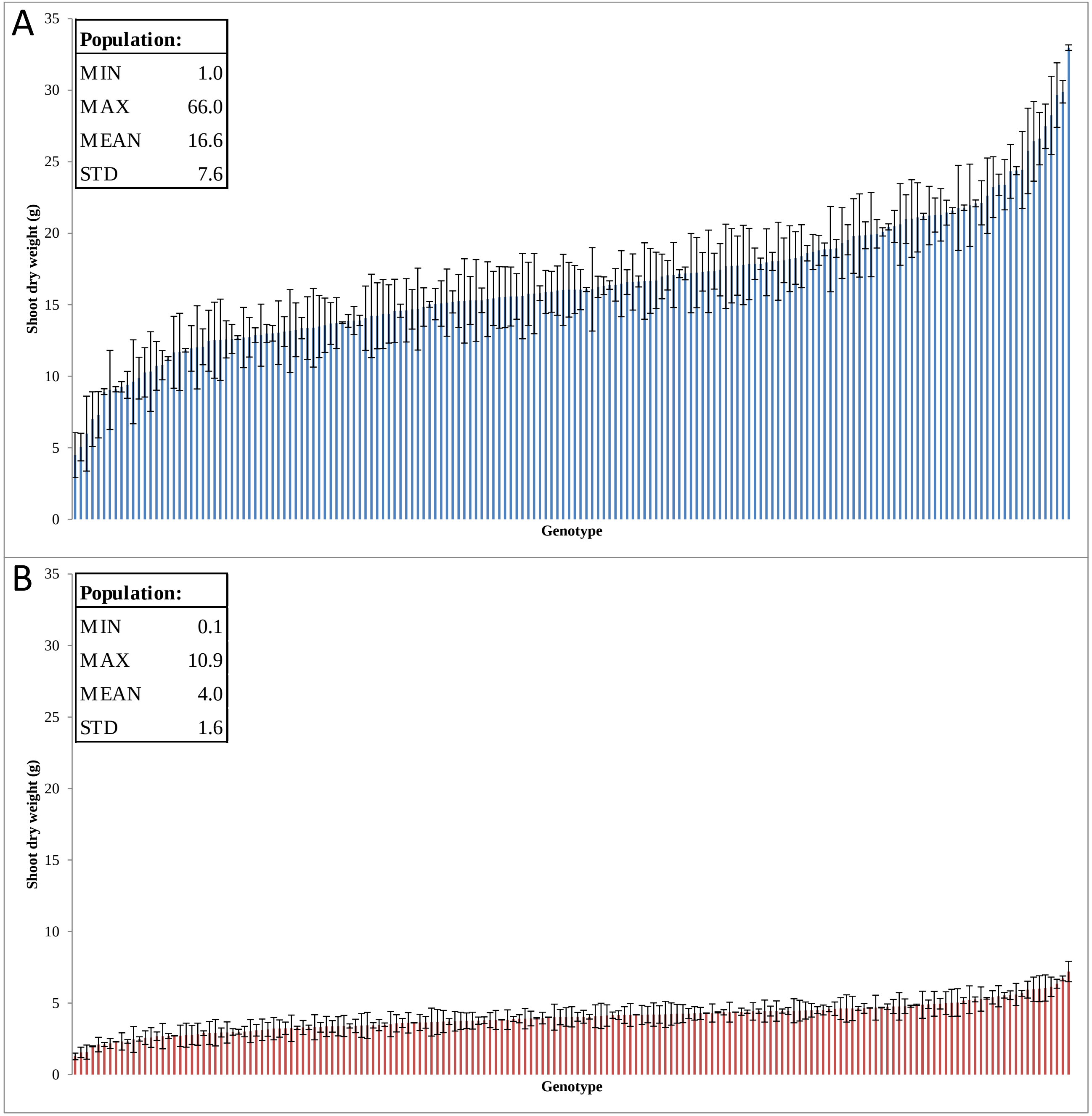

Figure S8: Phenotypic variation of shoot dry weight (Sdw) in 2015 of global population under control and drought conditions. A) Sdw (g) of genotypes of barley population under control conditions as well as population mean, max, min and standard deviation (STD). B) Sdw (g) of genotypes of barley population under drought conditions as well as population mean, max, min and standard deviation (STD). N = 4

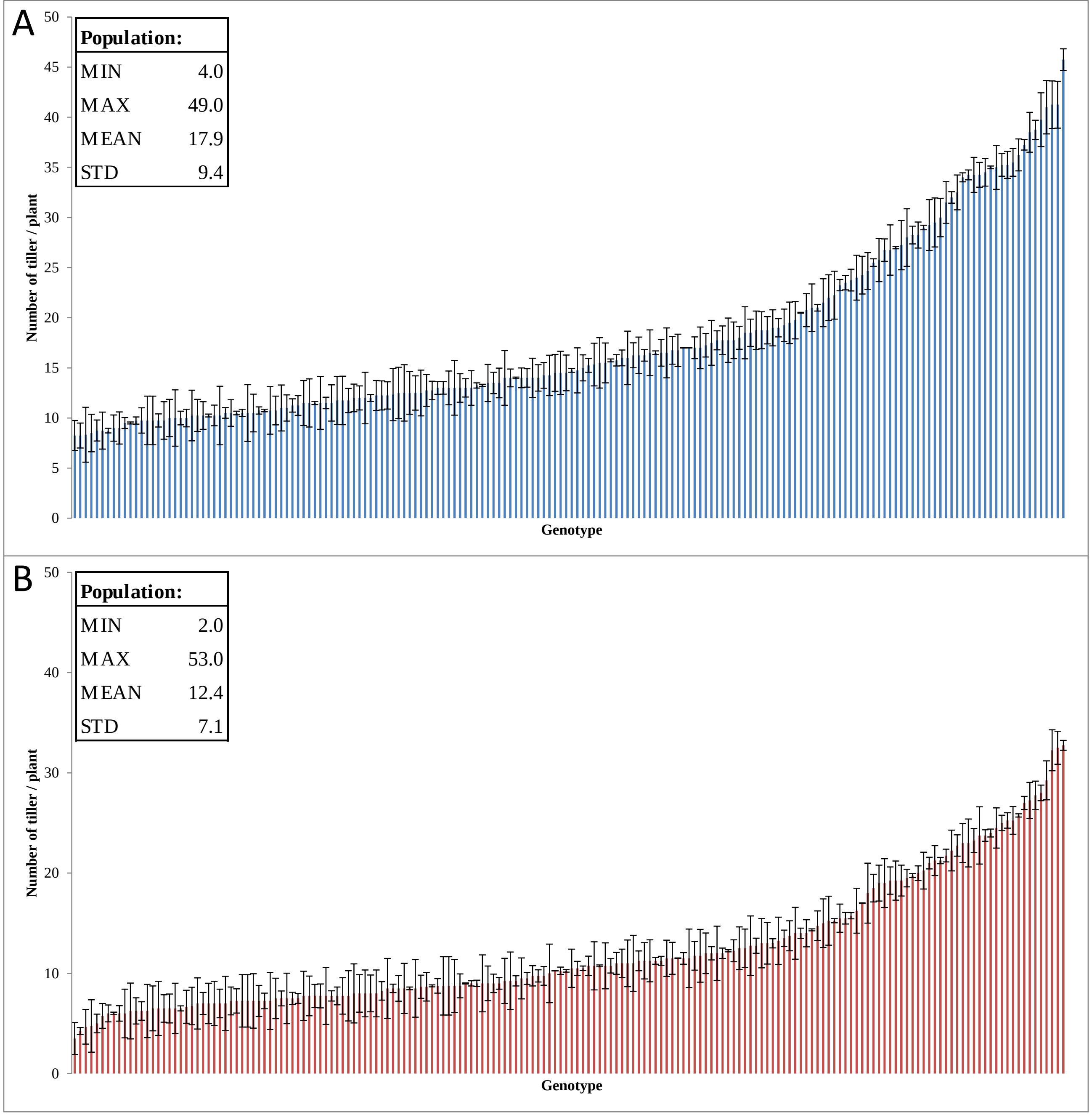

Figure S9: Phenotypic variation of tiller number (Til) in 2014 of global population under control and drought conditions. A) Til of genotypes of barley population under control conditions as well as population mean, max, min and standard deviation (STD). B) Til of genotypes of barley population under drought conditions as well as population mean, max, min and standard deviation (STD). N = 4

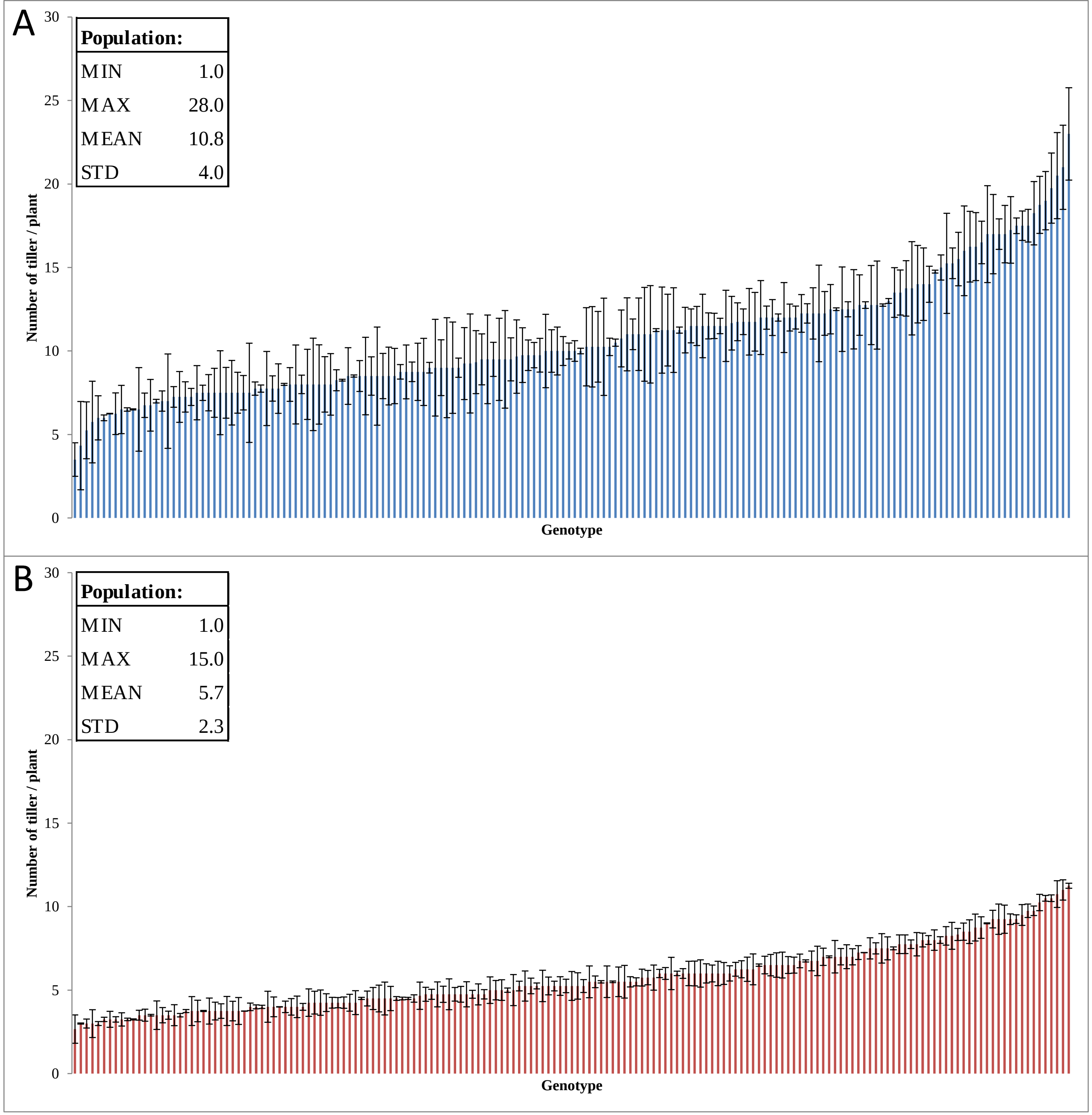

Figure S10: Phenotypic variation of tiller number (Til) in 2015 of global population under control and drought conditions. A) Til of genotypes of barley population under control conditions as well as population mean, max, min and standard deviation (STD). B) Til of genotypes of barley population under drought conditions as well as population mean, max, min and standard deviation (STD). N = 4

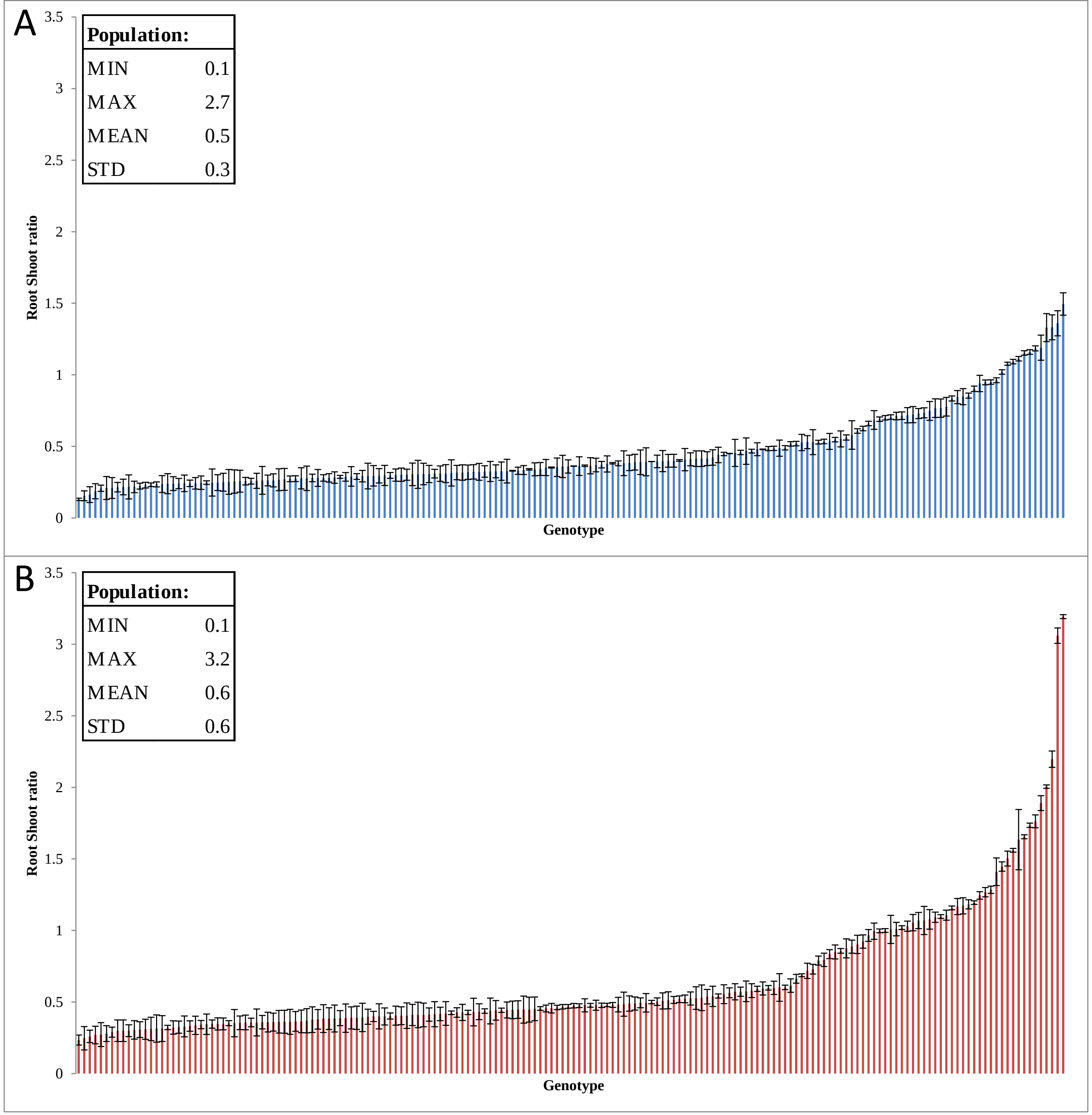

Figure S11: Phenotypic variation of root-shoot ratio (RS) in 2014 of global population under control and drought conditions. A) RS of genotypes of barley population under control conditions as well as population mean, max, min and standard deviation (STD). B) RS of genotypes of barley population under drought conditions as well as population mean, max, min and standard deviation (STD). N = 4

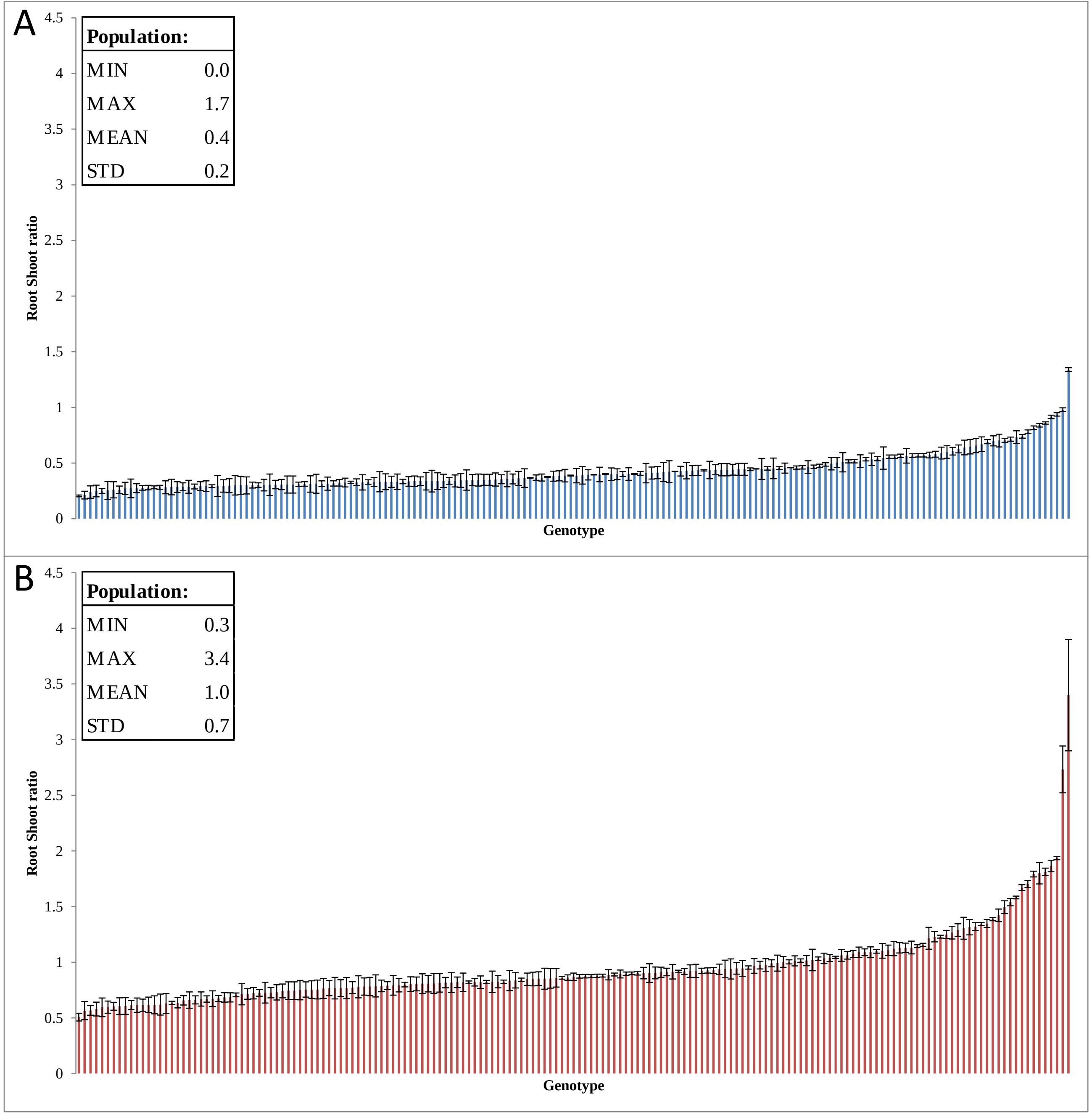

Figure S12: Phenotypic variation of root-shoot ratio (RS) in 2015 of global population under control and drought conditions. A) RS of genotypes of barley population under control conditions as well as population mean, max, min and standard deviation (STD). B) RS of genotypes of barley population under drought conditions as well as population mean, max, min and standard deviation (STD). N = 4

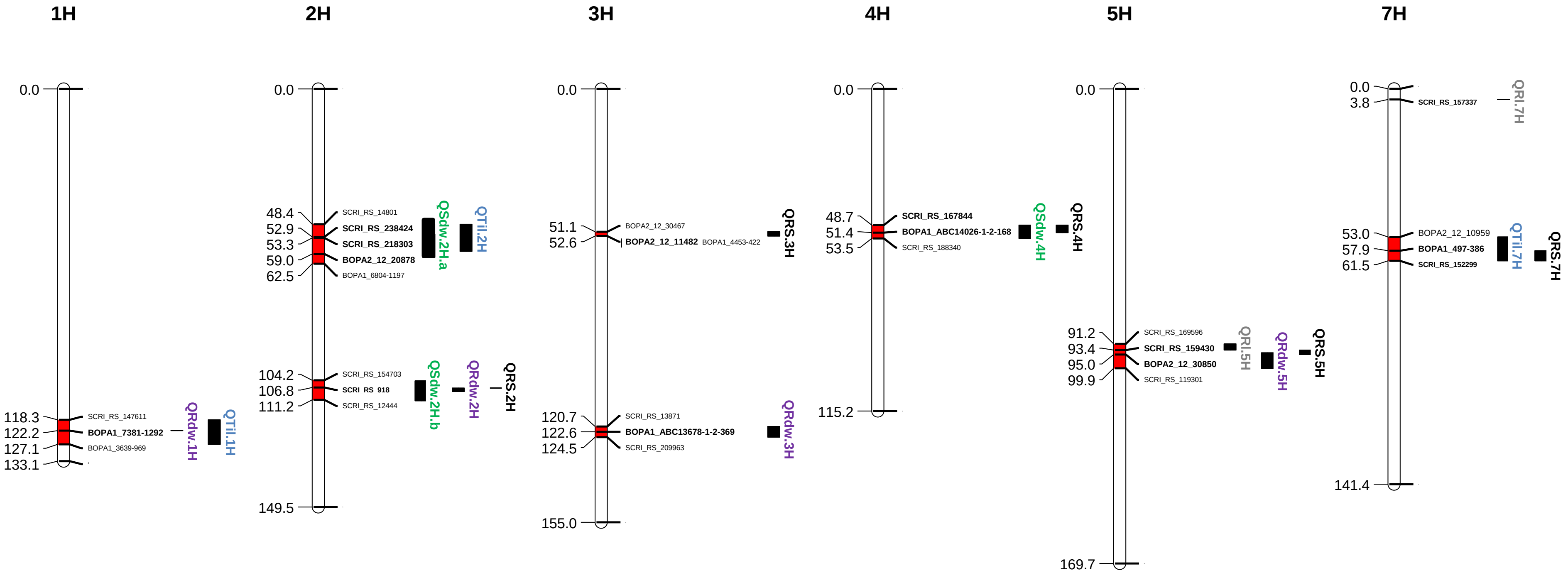

Figure S13: Association mapping for five different root and shoot traits on global diversity panel in molecular linkage map. 17 QTL located on six different chromosomes (1H, 2H, 3H, 4H, 5H and 7H). Purple: Root dry weight (Rdw); Grey: Root length (Rl); Green: Shoot dry weight (Sdw); Blue: Tiller number (Til); Black: Root-shoot ratio (RS). Flaking regions of QTL indicated in red and black bars.

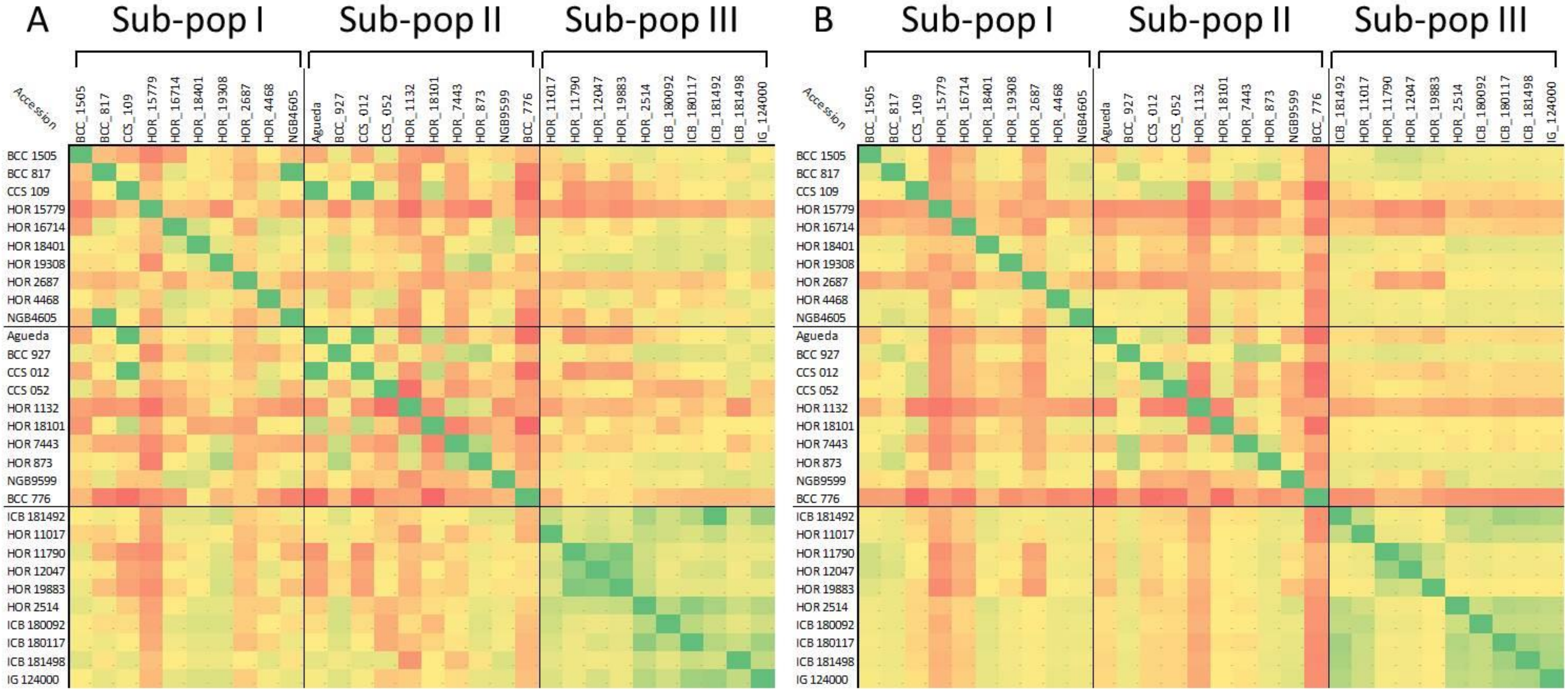

Figure S14: Genetic comparison of local and global genomic groups for QRdw.5H. Each group contains 30 randomly selected genotypes from the global barley population. A) Comparison of local genomic groups. B) Comparison of global genomic groups. Dark green: Rogers distance coefficient of 1.00, dark red: Rogers distance coefficient of 0.00

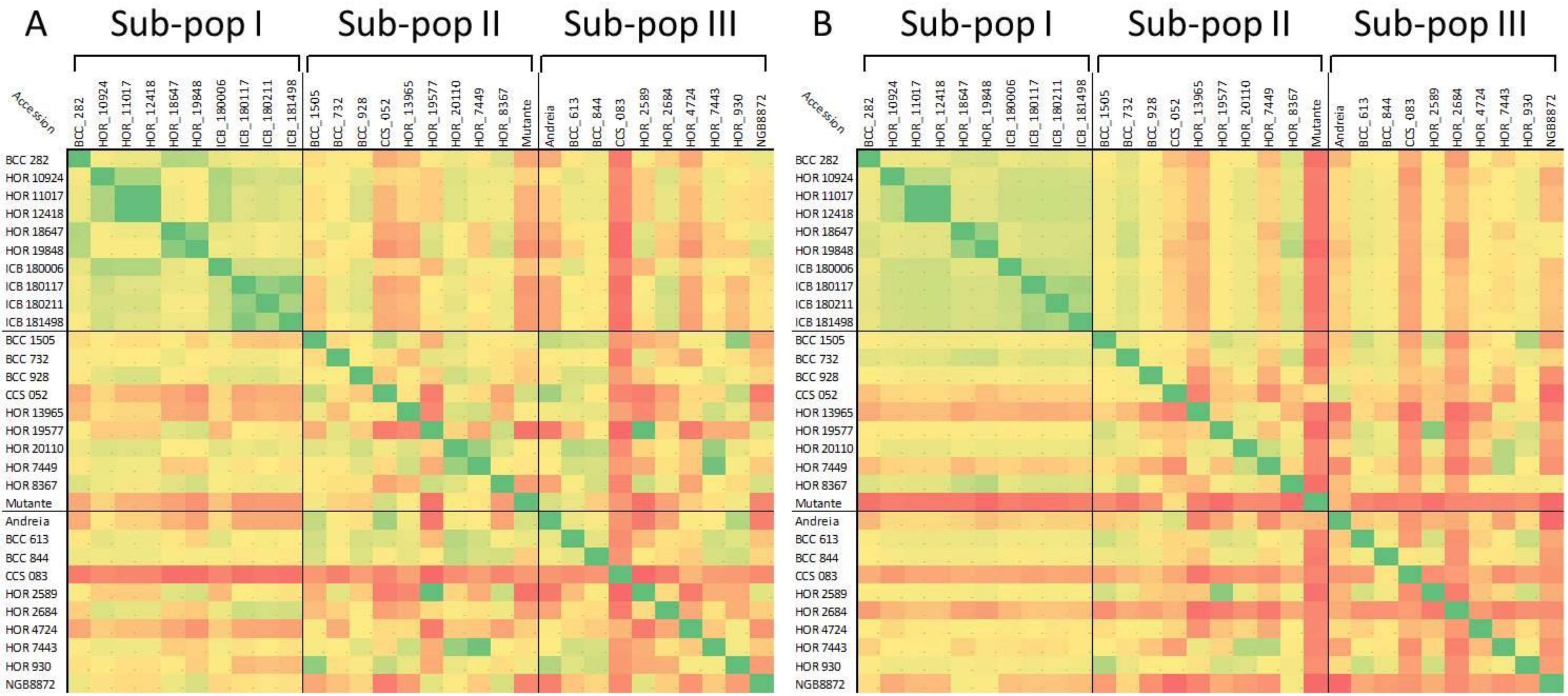

Figure S15: Genetic comparison of local and global genomic groups for QSdw.2H.b. Each group contains 30 randomly selected genotypes from the global barley population. A) Comparison of local genomic groups. B) Comparison of global genomic groups. Dark green: Rogers distance coefficient of 1.00, dark red: Rogers distance coefficient of 0.00

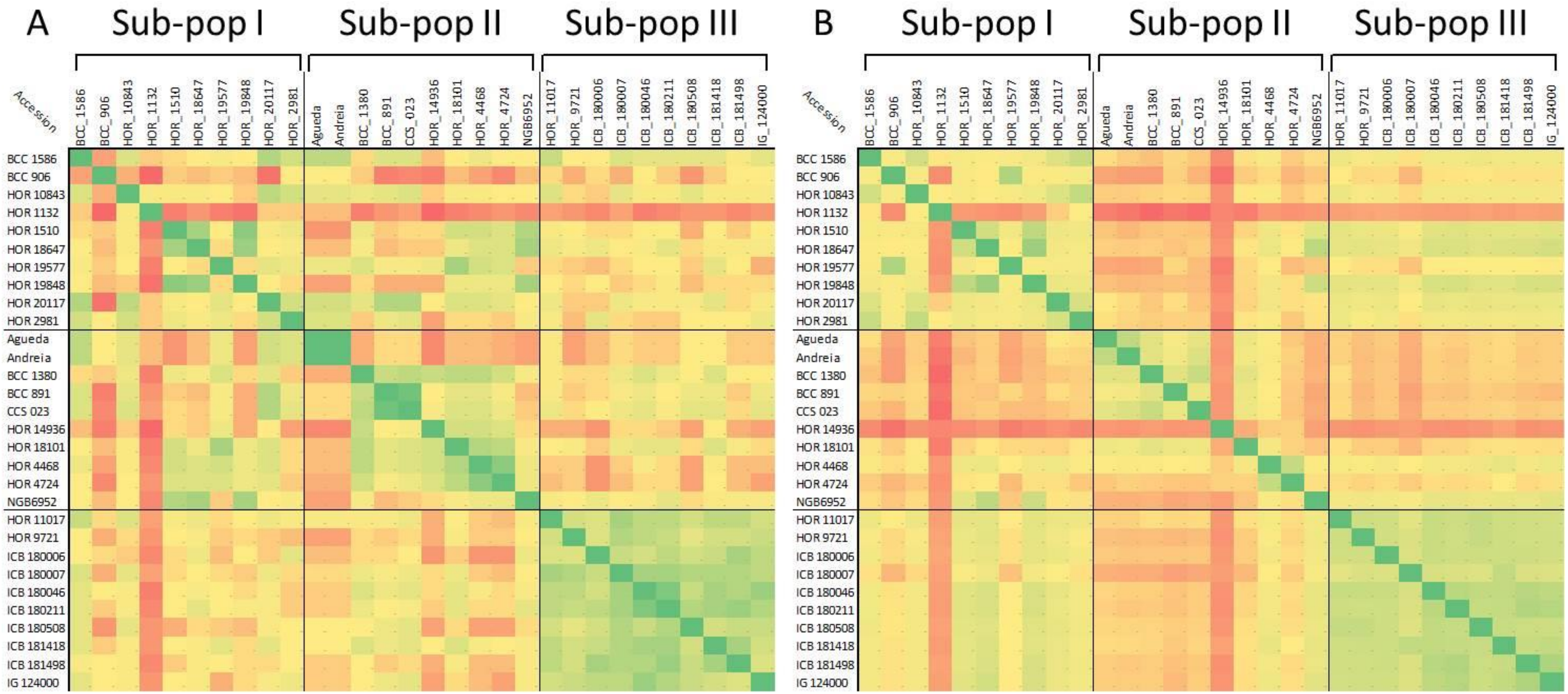

Figure S16: Genetic comparison of local and global genomic groups for QTil.1H. Each group contains 30 randomly selected genotypes from the global barley population. A) Comparison of local genomic groups. B) Comparison of global genomic groups. Dark green: Rogers distance coefficient of 1.00, dark red: Rogers distance coefficient of 0.00

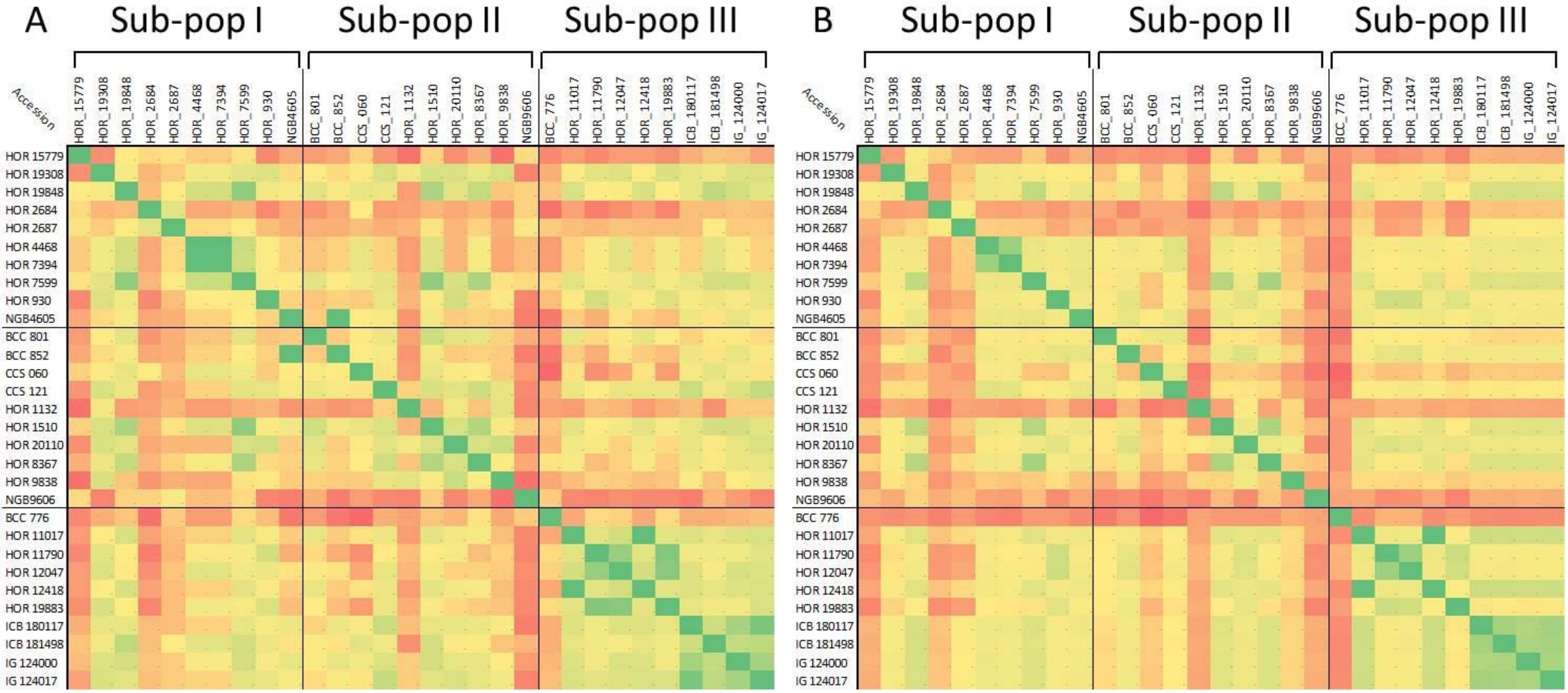

Figure S17: Genetic comparison of local and global genomic groups for QRS.5H. Each group contains 30 randomly selected genotypes from the global barley population. A) Comparison of local genomic groups. B) Comparison of global genomic groups. Dark green: Rogers distance coefficient of 1.00, dark red: Rogers distance coefficient of 0.00

```
                                                      +36
Sloop         atggaggacgcggcggccacgctggcgacggagctagacgggctgctggccatggcgagg
BCC906        atggaggacgcggcggccacgctggcgacggagctagacgggctgctggccatggcgagg
ICB180006     atggaggacgcggcggccacgctggcgacggagctggacgggctgctggccatggcgagg
              ***********************************.************************

Sloop         gagctggaggcgcgcgtcgacggcgaccagggcgcgcccggcgccgccagggagctctgc
BCC906        gagctggaggcgcgcgtcgacggcgaccagggcgcgcccggcgccgccagggagctctgc
ICB180006     gagctggaggcgcgcgtcgacggcgaccagggcgcgcccggcgccgccagggagctctgc
              ************************************************************

Sloop         tccgcgctggccgcgtccgtcgaccgggccgtgcgcctcgccggccgtggccgcaatgcc
BCC906        tccgcgctggccgcgtccgtcgaccgggccgtgcgcctcgccggccgtggccgcaatgcc
ICB180006     tccgcgctggccgcgtccgtcgaccgggccgtgcgcctcgccggccgtggccgcaatgcc
              ************************************************************

Sloop         ggcgccagggcgggcgtgaacggccagctcaggagcggcaggaaggcggcagcggcgaag
BCC906        ggcgccagggcgggcgtgaacggccagctcaggagcggcaggaaggcggcagcggcgaag
ICB180006     ggcgccagggcgggcgtgaacggccagctcaggagcggcaggaaggcggcagcggcgaag
              ************************************************************

Sloop         gtgcgcacgcaggtgcgggtggcctcaatgcaggacctcgggccactcgacgacgggatg
BCC906        gtgcgcacgcaggtgcgggtggcctcaatgcaggacctcgggccactcgacgacgggatg
ICB180006     gtgcgcacgcaggtgcgggtggcctcaatgcaggacctcgggccactcgacgacgggatg
              ************************************************************

Sloop         agctggaggaagtacggccagaagggcatcctcggcgccacgtacccgaggtcctacttc
BCC906        agctggaggaagtacggccagaagggcatcctcggcgccacgtacccgaggtcctacttc
ICB180006     agctggaggaagtacggccagaagggcatcctcggcgccacgtacccgaggtcctacttc
              ************************************************************

Sloop         cggtgcacgcaccggcacacgcagggctgcgccgccaccaagcaggtgcagcgcgcgacg
BCC906        cggtgcacgcaccggcacacgcagggctgcgccgccaccaagcaggtgcagcgcgcgacg
ICB180006     cggtgcacgcaccggcacacgcagggctgcgccgccaccaagcaggtgcagcgcgcgacg
              ************************************************************
                                            +451
Sloop         gccgacccgctgctcttcgacgtcgtgtacgtgggcgcgcacacctgcgccggggccgcg
BCC906        gccgacccgctgctcttcgacgtcgtgtacgtgggcgcgcacacctgcgccggggccgcg
ICB180006     gccgacccgctgctcttcgacgtcgtgtacttgggcgcgcacacctgcgccggggccgcg
              ****************************** *****************************
                                                    +515
Sloop         gtcctgggcggcacggagcagcagctgccgccgccctgctgccttcggccaggagcagcag
BCC906        gtcctgggcggcacggagcagcagctgccgccgccctgctgccttcggccaggagcagcag
ICB180006     gtcctgggcggcacggagcagcagctgccgccgcttgctgccttcggccaggagcagcag
              **********************************.*************************
```

Figure S18: Putative WRKY29 transcription factor in different barley accessions. Nucleotide alignment of putative *HvWRKY29* in cultivated barley Sloop (DQ863113) as well as BCC906 and wild barley ICB180006 was made using MAFFT. The gray tag indicates polymorphisms. “*” indicates the identical nucleotides in all sequences. “:” indicates conserved substitutions. “.” indicates semi-conserved substitutions. “ “ indicates non-conserved substitutions.

```
Optic         atggacatgggcgaggtctcgagctctccccctcctcttccaacgagaacgcgtcggga
HOR4206       atggacatgggcgaggtctcgagctctccccctcctcttccaacgagaacgcgtcggga
ICB180006     atggacatgggcgaggtctcgagctctccccctcctcttccaacgagaacgcgtcggga
              ************************************************************
                                                 +96
Optic         cggtcgtcgacggccaagcgcccggcggggcgcactaagttccgcgagacaaggcacccg
HOR4206       cggtcgtcgacggccaagcgcccggcggggcgcactaagttccgcgagacaaggcacccg
ICB180006     cggtcgtcgacggccaagcgcccggcggggcgcaccaagttccgcgagacaaggcacccg
              ***********************************.************************

Optic         gtgtaccgcggcgtgcggcgccggggcaacgccgaacggtgggtatgcgaggtgcgcgtc
HOR4206       gtgtaccgcggcgtgcggcgccggggcaacgccgaacggtgggtatgcgaggtgcgcgtc
ICB180006     gtgtaccgcggcgtgcggcgccggggcaacgccgaacggtgg------------------
              ******************************************

Optic         cccggcaagcgcggcgctcggctctggctcgggacttacgccacggccgagatcgcagcg
HOR4206       cccggcaagcgcggcgctcggctctggctcgggacttacgccacggccgagatcgcagcg
ICB180006     ------------------------------------------------------------

Optic         cgcgcgaacgatgccgcaatgctcgccctggcgtccgctccgccgcgcggctcaacttc
HOR4206       cgcgcgaacgatgccgcaatgctcgccctggcgtccgctccgccgcgcggctcaacttc
ICB180006     ---------------------------------gtccgctccgccgcgcggctcaacttc
                                               ***************************

Optic         ccggactccgcgtggctgctcgccgtgccgtccgcgcactccgatctcgccgacgtccgg
HOR4206       ccggactccgcgtggctgctcgccgtgccgtccgcgcactccgatctcgccgacgtccgg
ICB180006     ccggactccgcgtggctgctcgccgtgccgtccgcgcactccgatctcgccgacgtccgg
              ************************************************************

Optic         cgcgcggcggtcgaggccgtcgcggacttgcagcgacgggaggccgccggtgggtccatc
HOR4206       cgcgcggcggtcgaggccgtcgcggacttgcagcgacg----------------------
ICB180006     cgcgcggcggtcgaggccgtcgcggacttgcagcgacgggaggccgccggtgggtccatc
              **************************************

Optic         accgccacagtcggggaggaggcctcccgtggcgctcctgcagaatcgtcgtctgagtct
HOR4206       -----------------ggaggcctcccgtggcgctcctgcagaatcgtcgtctgagtct
ICB180006     accgccacagtcggggaggaggcctcccgtggcgctcctgcagaatcgtcgtctgagtct
                               *******************************************

Optic         gaggatgccggttcgtcggagacgtcgaaaccttccgccggtggagacttggcggtaccg
HOR4206       gaggatgccggttcgtcggagacgtcgaaaccttccgccggtggagacttggcggtaccg
ICB180006     gaggatgccggttcgtcggagacgtcgaaaccttccgccggtggagacttggcggtaccg
              ************************************************************

Optic         gtcggaatggacgtcgaaatgttcaggcttgacttcttcccggaaatggagtttggctcg
HOR4206       gtcggaatggacgtcgaaatgttcaggcttgacttcttcccggaaatggagtttggctcg
ICB180006     gtcggaatggacgtcgaaatgttcaggcttgacttcttcccggaaatggagtttggctcg
              ************************************************************
                                           +630
Optic         tactacgcgagcctcgcggaggcgctgctcatggacccgccgccggtggcgaacagcacg
HOR4206       tactacgcgagcctcgcggaggcgctgctgatggacccgccgccggtggcgaacagcacg
ICB180006     tactacgcgagcctcgcggaggcgctgctgatggacccgccgccggtggcgaacagcacg
              ***************************** ******************************
```

Figure S19: CBF10B transcription factor in different barley accessions. DNA alignment of *HvCBF10B* in cultivated barley Optic (AAX28956) and HOR4206 as well as wild barley ICB180006 was made using MAFFT. Polymorphisms are indicated by a light gray tag. Consensus symbols donated as: "*" indicates the identical nucleotide in all sequences. "." indicates semi-conserved substitutions. " " indicates non-conserved substitutions.

```
                                                                          +53
Nure            atggacatgggccttgaggtctcgagctctcccccctcctcttccaacgagaacgcgtcg
HOR4206         atggacatgggccttgaggtctcgagctctcccccctcctcttccaacgagaacgcgtcg
ICB180006       atggacatgggccttgaggtctcgagctctcccccctcctcttccaacgagatcgcgtcg
                **************************************************** *******

Nure            ggacggtcgtcgacggccaagcgcccggcggggcgcaccaagttccgcgagacaaggcac
HOR4206         ggacggtcgtcgacggccaagcgcccggcggggcgcaccaagttccgcgagacaaggcac
ICB180006       ggacggtcgtcgacggccaagcgcccggcggggcgcaccaagttccgcgagacaaggcac
                ************************************************************
                                                               +168     +177
Nure            ccggtgtaccgcggcgtgcggcgccggggcaacgccgaacggtgggtatgcgaggtgcgc
HOR4206         ccggtgtaccgcggcgtgcggcgccggggcaacgccgaacggtgggtctgcgaggttcgc
ICB180006       ccggtgtaccgcggcgtgcggcgccggggcaacgccgaacggtgggtctgcgaggttcgc
                *********************************************** ******** ***
                                                      +219
Nure            gtccccggcaagcgcggcgctcggctctggctcgggacttacgccacggccgagatcgca
HOR4206         gtccccggcaagcgcggcgctcggctctggctcgggacgtacgccacggccgagatcgca
ICB180006       gtccccggcaagcgcggcgctcggctctggctcgggacgtacgccacggccgagatcgca
                ************************************** *********************
                           +252                                          +294
Nure            gcgcgcgcgaacgatgccgcaatgctcgccctgggcggccgctccgccgcgcgcctcaac
HOR4206         gcgcgcgcgaacgatgccgcaatgctcgccctgggcggccgctccgccgcgcgcctcaac
ICB180006       gcgcgcgcgaatgatgccgcaatgctcgccctgggcggccgctccgccgcgcggctcaac
                ***********.*****************************************.******
                   +304
Nure            ttctcggactccgcgtggctgctcgccgtgccgtccgcgcactccgatctcgccgacgtc
HOR4206         ttctcggactccgcgtggctgctcgccgtgccgtccgcgcactccgatctcgccgacgtc
ICB180006       ttcccggactccgcgtggctgctcgccgtgccgtccgcgcactccgatctcgccgacgtc
                ***.********************************************************
```

Figure S20: CBF10A transcription factor in different barley accessions. DNA alignment of *HvCBF10A* in cultivated barley Nure (DQ445241) and BCC906 as well as wild barley ICB180006 was made using MAFFT. Polymorphisms are indicated by a gray tag. Consensus symbols donated as: "*" indicates the identical nucleotide in all sequences. "." indicates semi-conserved substitutions. " " indicates non-conserved substitutions.

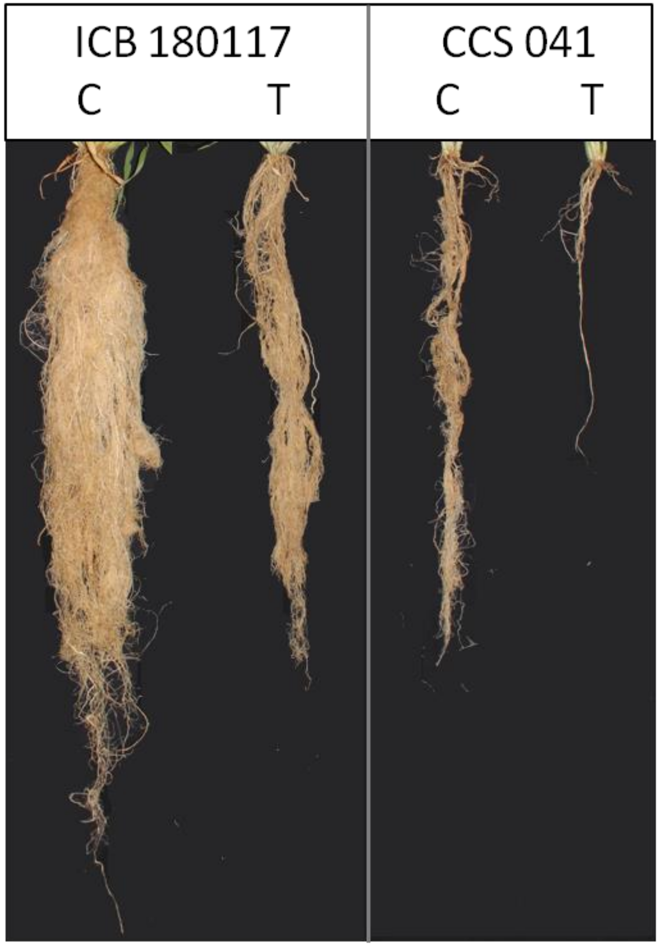

Figure S21: Root mass of two extreme barley genotypes under drought (T) and control (C) conditions. ICB180117 wild barley accession from Israel and CCS 041 modern cultivar from Germany.
